# Italian Consensus Statement on Patient Engagement in Chronic Care: Process and Outcomes

**DOI:** 10.3390/ijerph17114167

**Published:** 2020-06-11

**Authors:** Guendalina Graffigna, Serena Barello, Giuseppe Riva, Massimo Corbo, Gianfranco Damiani, Primiano Iannone, Albino Claudio Bosio, Walter Ricciardi

**Affiliations:** 1 EngageMinds Hub – Consumer, Food and Health Engagement Research Center, Department of Psychology, Università Cattolica del Sacro Cuore, 20123 Milan, Italy; giuseppe.riva@unicatt.it (G.R.); claudio.bosio@unicatt.it (A.C.B.); 2 Department of Psychology, Catholic University of Milan, 20123 Milan, Italy; 3 Casa di Cura Privata del Policlinico, 20100 Milan, Italy; m.corbo@ccppdezza.it; 4 Fondazione Policlinico Universitario A., Gemelli IRCCS, 00168 Roma, Italy; gianfranco.damiani@unicatt.it (G.D.); walter.ricciardi@unicatt.it (W.R.); 5 Faculty of Medicine and Surgery, Università Cattolica del Sacro Cuore, 00168 Roma, Italy; 6 Higher Institute of Health, 00168 Rome, Italy; primiano.iannone@iss.it

**Keywords:** patient engagement, digital technologies, recommendation, consensus conference, guidelines, health services research, chronic care

## Abstract

Patient engagement has been recognized as a key priority in chronic care. However, scholars agree that guidelines are needed to ensure effective patient engagement strategies. To this end, a Consensus Conference process was promoted with the following methodological steps: (1) extensive literature review about patient engagement initiatives in chronic care; (2) a stakeholders survey to collect best practices and (3) workshops with experts. On the basis of the information collected, a consensus statement was drafted, revised, and finalized by a panel of select renowned experts. These experts agreed in defining engagement as an eco-systemic concept involving multiple actors all of which contribute to influence patients’ willingness and ability to engage in chronic care. Moreover, experts recommended, whenever possible, to adopt standardized instruments to assess engagement levels and related unmet needs. Then, experts strongly advised appropriate trainings for healthcare professionals about patient engagement strategies. Furthermore, the importance of promoting healthcare professionals’ wellbeing has been advocated. Family caregivers, as well as patients’ organizations - should be trained and engaged to increase the effectiveness of interventions dedicated to patients. Finally, experts agreed that digital technologies should be considered as a crucial enhancer for patient engagement in chronic care.

## 1. Introduction

The concept of patient engagement has gained increasing attention in health care in both the clinical and research field [1,2,3,4,5,6,7,8,9,10,11,12,13,14,15,16]. Recent decades, indeed, have witnessed a profound shift of care models towards an increasing focus on the role of the patient, who is seen as an active and “expert” subject within the clinical care process who is willing to co-manage his/her own health [17,18,19,20,21]. Furthermore, patients and their family members express the desire to acquire a more active role in all the phases of their healthcare journey [22,23,24,25,26,27,28,29]. Patients want to be more involved in the decision-making process related to their health care; they want to be thoroughly aware about all the possible treatment options, and the relative pros and cons [30,31,32,33,34,35,36]. Even if researchers and clinicians agree with the ethical and pragmatic priority of patient engagement’s promotion, at present there is no consensus regarding the definition of patient engagement and the more recommended strategies to reach it. Moreover, patients’ and healthcare professionals’ perspectives on the factors that could support or hinder patient engagement have not been sufficiently studied yet, and there are still no recommendations regarding o the most effective interventions in fulfilling patient engagement.

The Università Cattolica del Sacro Cuore (Milan, Italy) in collaboration with the Welfare General Directorate of Lombardy Region (Northern Italy), and under the methodological supervision of the Italian National Institute of Health (ISS), promoted a Consensus Conference to agree on recommendations for Patient Engagement in chronic care.

The Consensus Conference represented the occasion to promote a multidisciplinary and cross-disease discussion among experts (see Section 2.1) to seek answers to the following questions:

1) How to define patient engagement in chronic care?
2) How can patient engagement be measured?
3) What are the most recommended methodologies and tools to promote patient engagement?
4) What is the role of new technologies in promoting of patient engagement?

## 2. Methods

A comprehensive, multi-stakeholder approach (described in the next paragraphs) was used to develop a consensus statement on patient engagement in chronic care according to the standard procedure defined by the Consensus Development Program of the United States’ NIH which, since 1977, has been developing standards and guidelines to assess and reach consensus on controversial medical issues.

This project occurred between November 2016 and June 2017 and included: (1) a wide *literature review* on published evidence about patient engagement in chronic care (see Table 1); (2) a *stakeholders survey* to collect best practices and experiences to promote patient engagement in the Italian chronic care setting; (3) four *workshops with experts and patients representatives* to discuss and rate the evidences collected through the literature scan and the stakeholders’ survey; (4) an expert panel to finalize *the Consensus Statement* by reviewing, rating, taking decisions and making recommendations related to the final document of consensus statement.

The research team coordinated the finalization of the Consensus Statement document thanks to ongoing direction and support along the process provided by methodologist experts in Consensus Development Processes belonging to the Higher Institute of Health of Italy. The Consensus Statement document was finalized, designed, and disseminated to public opinions and experts throughout the Higher Institute of Health of Italy website, the website of Lombardy Region and, the website of Università Cattolica and thanks to the sharing of these online links by all the experts and institutions involved in the project. Throughout the entire Consensus Conference process, patients and family caregivers’ representatives, selected by the patients’ associations participating in the project, were closely involved to raise their voices and negotiate, together with the other representatives, on their expectations and priorities for promoting patient engagement. The research team paid close attention to ensure the equity of participation of patients and caregivers representatives in all the phases of the methodological processes, by moderating power dynamic with other experts and by ensuring adequate space for everyone to speak out both in the stakeholders survey, in the workshops to discuss the collected evidences and in the experts’ panel (for instance, the research team guaranteed a leading position (vice-president) for a patient representative on the experts’ panel).

### 2.1. Participants

The Consensus Conference involved 104 experts in patient engagement promotion from diverse scientific and professional backgrounds (i.e., medicine, psychology, sociology, nursing, healthcare management, public health, policy making, health engineering) and from the scope of patient advocacy and voluntary organizations. This in order to promote a broad, trans-disciplinary and cross-disease debate and to reach a stronger consensus. Expert was defined as somebody who possesses relevant knowledge, skills and experience, and whose opinion is accepted by other persons in the field of affiliation. In order to guarantee the scientific standards of the process the stakeholders involved in the project were asked to sign a conflict of interest statement and to accept the Consensus conference regulation.

In Table 2, a synthetic description of the background of experts involved in the process is reported. The whole list of experts in reported in Appendix A.

### 2.2. Literature Review

An extensive scan of the international scientific literature published in English was conducted for the topics object of the consensus conference using the following databases: Cochrane Library, Web of Science, PubMed, Scopus, CINAHL, PsychInfo. In particular, the two different reviews were performed according to the different topics in analysis (see Table 1 for a detailed description of the literature review processes):

For queries 1 and 2 a narrative review of literature was conducted in the aim of exploring the nature and characteristics of concepts, theories and definitions of Patient Engagement. The analysis followed the principles of Conceptual Analysis, widely acknowledged in both social and medical sciences [12,39,41] and was conducted by a group of researchers trained in narrative review (GG, SB). The conceptual analysis conducted was inspired by Walker and Avant’s [38] methodological principles, who have systematized an inductive process of content analysis for mapping and synthetizing the main themes and conceptual content addressed by the relevant literature contributions selected in the analysis. In particular, a thorough qualitative analysis of the sources found was conducted through an iterative process of comparison between the definitions found and the continuous critical validation of the concepts emerging during the analysis process itself. This iterative and interpretative process of analysis allowed to clarify the theoretical roots of the conceptualizations of patient engagement thanks to the continuous comparison and differentiation process between the definitions found in literature, the description of their characteristics and peculiarities, the definition of their main attributes, as well as the overlapping areas with other concepts. More in detail, the analysis was finalized by highlighting and classifying the attributes, the conceptual antecedents and consequences as well as means of empirical operationalization of the concept of Patient Engagement as proposed by the definitions and the theoretical models found in literature [8].

For queries 3 and 4, was conducted a systematic literature review according to the methodological principles of Preferred Reporting Items for Systematic Reviews and Meta-Analyses (PRISMA) [42], which represent a golden standard of the international guidelines for conducting systematic reviews of scientific literature. According to this approach, the selected sources underwent systematic analysis with the aim of extracting the following information in a structured analysis grid: (1) methodological characteristics of the study (country of the first author, study design, number of branches of the study, type of control group, n. of participants in the experimental and control group) (2) participants’ characteristics (diagnosis, average age, % per gender); (3) characteristics of the intervention (number and duration of the sessions, type of treatment, strategies and instruments used both for groups and single individuals, theoretical foundations of the intervention); (4) obtained results (measured outcomes, methods of evaluation of the results, results achieved overall). The data extracted was then qualitatively analyzed. Evaluation of the quality of the studies: The quality of the RCT studies was evaluated following the Downs and Black checklist, a solid instrument with good psychometric properties [39] that has high levels of correlation with other instruments of quality assessment study [40].

The methodological quality was reviewed and assessed by methodologists belonging to the Italian Higher Institute of Health (and included among this paper authors: PI and AF) experts in scientific literature analysis and in the methodological standards required by the Consensus Development Process Methodological Manual.

### 2.3. Stakeholder Survey

An ad hoc survey with open-ended questions (i.e., please, describe the patient engagement intervention characteristics, its target, the setting of application, obtained results and its potential transferability) was used to collect input from stakeholders on good practices, instruments and methodologies used for the promotion of patient engagement. This online survey aimed at gathering the experiences of experts on their initiatives and practices for promoting patient engagement in chronic care in Italy. The survey was self-administered online throughout the Qualtrics platform dedicated to web-survey (a dedicated platform for delivering online survey broadly used in academia). Respondents were asked to report any valuable initiatives aimed at promoting patient engagement by describing in details their characteristics (i.e., intervention aims, settings, main target, instruments, outcomes measures, innovativeness and transferability…). The stakeholder survey was disseminated by the research team to their networks across Italy, reaching many stakeholders in the clinical and public health/health promotion sectors.

The survey involved a sample of 104 experts that was not statistically representative but meaningful for the scope of this investigation (see Section 2.1). The experts were selected on the basis of a snowball sampling process which is a nonprobability sampling technique where the experts initially included in the Consensus Development Process were asked to recruit future subjects among their professionals or social networks basing on the inclusion criteria of being an expert in patient engagement (see Section 2.1). The survey took place between March and April in 2017. Due to the qualitative nature of data collected, data were analyzed by thematic analysis and were summarized in written reports to be discussed in the workshops with experts (see Section 2.4) as materials useful for drafting the final consensus statement.

### 2.4. Workshops with Experts

Four face-to-face workshops with a section of the stakeholder (ten experts each group) who participated to the survey were conducted in order to synthetize, discuss and enrich the evidence collected from the literature scan and the stakeholders’ survey. All workshops involved a balanced mix of professionals belonging to different disciplinary backgrounds and expertise in order to guarantee the maximum variety of perspectives in the discussion. Furthermore, all workshops involved patients’ and caregivers’ representatives (see Table 2 for a detailed description of the disciplinary field of experts involved in the workshops). Selection of stakeholders was based on their availability and willingness to participate in the face-to-face workshop and by the criterion of guaranteeing the multi-disciplinary balance of experts involved in each group, in order to allow a rich discussion moving from different perspectives. Of the four workshops, one was focused on definition of patient engagement in chronic care; one on the tools to measure it; one on the intervention to promote it and one on digital tools for patient engagement).

Workshops lasted about four hours each and were moderated in a non-directive way by two researchers (G.G. and S.B.) experts in qualitative research who take care that all the participants had equal chance to give their input to the discussion. Particular attention was payed to guarantee that patients could have space to speak out in the process. Transcript of the workshops’ discussions underwent a qualitative thematic analysis made by the two moderators in order to synthetize the main topics emerged. Furthermore, the four reports emerged from the workshops’ transcript analysis were revised and finally approved by all workshops’ participants throughout an iterative email exchange. The final agreed version of the workshop reports was passed to the experts’ for an ulterior phase of discussion, enrichment and consensus.

### 2.5. Finalization of the Consensus Statement

Finally, a panel had to revise and rate the reports produced by the workshops with experts to finalize the consensus statement. The experts’ panel was composed of members selected by the research team according to the following criteria: having relevant knowledge and experience, whose opinion being accepted by others in the field of affiliation (e.g.,: relevant journal publications, published books, presentations at conferences, etc.), belonging to different disciplinary field, and being patients representative with long and national wide experience in patient advocacy and in promoting initiatives of patient engagement. The panel was in charge of revising and evaluating the report produced and approved from the experts’ workshops. The panel also discussed, drafted and approved the final consensus statement (see Appendix A for a detailed description of the members included in the experts’ panel). All the members of the panel had to express their feedback on the consensus statement and approve its final version.

## 3. Results

In the following sub-sections, we provide a synthesis of the main evidences that emerged and the derived consensus statements. Additional scientific material has been published elsewhere [40,41,42].

### 3.1. Query 1 – How Can Patient Engagement be Defined?

#### 3.1.1. Main Evidence

**Figure 1:**
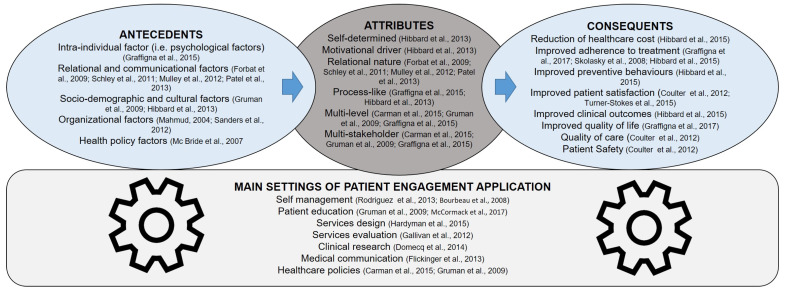
Antecedents, attributes, consequences and settings of application of patient engagement derived from the narrative review (see methodological section).

The definitions of patient engagement available in the literature (see Table 3) tend to differ in terms of attributes, antecedents, consequences and settings of application of this concept. However, despite this great variety, some features are common across these definitions (see Figure 1 for a synthesis). In particular, among the many definitions retrieved in the literature and according to the direct clinical experience of the experts engaged in the process, patient engagement emerges as a relational concept. This concept relates not only to patients behaviors [43] along their healthcare journey, but it also refers to the motivations and feelings which are linked to the patient engagement experience. Moreover, patient engagement emerged as a multi-actor process [44], meaning that it may develop and be maintained only within significant collaboration among actors (patients, healthcare professionals, caregivers, healthcare system managers, etc.) who all share a common goal (such as the achievement of good health outcomes). Thus, relationships and interactions to foster the process of patient engagement are several, at different organizational levels and should be all coordinated to enable the partnership between patients and the healthcare system as a whole. Namely engagement is related to the positive relation of the patient with his/her health and/or care condition (i.e., the individual’s level of psychological adjustment to the disease); with his/her informal caregiver (the family in particular) [4]; with his/her healthcare professionals [45]; with society, and so on. Due to this relational and interactive nature, patient engagement emerges as a systemic and organizational phenomenon resulting from a combination of multilevel factors of individual, relational, organizational, social and political nature. As a consequence, in terms of factors determining patient engagement (i.e., antecedents) there is agreement in the literature and among the interviewed experts that individuals may differ in their attitude towards the proposal of becoming more engaged in their healthcare journey. This may be due to their clinical or socio-cultural characteristics but also to the organizational and structural characteristics of the healthcare setting. Another important factor which effects on engagement levels is the degree of emotional elaboration (i.e., psychological adjustment) and adaptation to the illness and its management [46]. At the same time, the needs and attitudes of the professionals and the healthcare team towards the development of patient engagement are aspects that need just as much monitoring. Finally, crucial barriers to patient engagement are variously related to the organizational culture of health providers, institutional biases, and political environments: those are all elements that at the meso and macro level could impact on the engagement journey [45,47,48].

#### 3.1.2. Consensus Statement

Given its relational and multi-stakeholder nature, the consensus conference recommended to avoid the term “patient” in the final definition of the concept: engagement was considered not only a “fact pertaining to the patient”, but rather an experience related to the whole healthcare system and the different actors involved in it. Engagement, thus, should not be conceived only as a duty of the patients who should be more proactive in the medical journey. On the contrary, engagement is conceived as the coordinated and consensual course of action of all the different stakeholders at the different organizational levels. Patients can become truly engaged if healthcare professionals are engaged too, in recognizing the value of patients’ participation; if healthcare organizations are engaged and able to change their process and models in order to enable patient engagement; if society is truly engaged and supportive of a new approach to health and healthcare.

Moreover, the use of the word *patient* appeared incoherent to the consensus experts because it is excessively passivizing: the use of the term “patient” associated with the term “engagement”, implicitly evokes a negative and asymmetric relationship between an “expert” (i.e., the healthcare professional) and a “lay” person who must be changed to some extent. It thus sounds like a medicalizing approach to the concept which risks failing to be consistent with the ethical and pragmatic nature of the term itself. Furthermore, due to the relational and multi-actor nature of the engagement process discussed above, speaking only of “patients” appears to be a tokenistic reduction of the complexity and relational nature of the concept. Engagement is not only a patient-related issue; it is a phenomenon which may exist and develop only within the scope of a shared commitment of all the actors involved in the healthcare arena. As a consequence, the consensus conference recommended using the term “engagement” without pinning it to a specific actor (i.e., the patient) so as to underline the relational nature of the concept.

Moreover, the consensus experts recommended that engagement should be defined as a driving motivational and affective force which orients participative actions and which is fueled within the significant healthcare relationships taking place along the healthcare journey. Finally, the consensus experts defined engagement as a psychosocial process which is the base for enabling other clinical process and actions such as adherence, compliance, empowerment, activation, health literacy and shared decision making. The latter are clearly different in their nature from Engagement, but are often considered jointly both in the scientific literature and in the clinical practice.

### 3.2. Query 2 – How Can Patient Engagement be Measured?

#### 3.2.1. Main Evidence

Based on the narrative review conducted, patient engagement scientists agree on the importance of the inclusion of a systematic assessment and monitoring of engagement to orient the healthcare and welfare system planning as well as the care delivery [28,35]. Diverse validated and reliable measures of engagement exist in the literature, but they are focused on alternative components of that experience. Furthermore, while the most widely used engagement scales concern patients, some of them also assess healthcare professionals’ attitudes and behaviors towards patient engagement promotion and to family caregivers’ needs and experience of engagement in assisting their loved ones. To be included in the list, retrieved measures from the literature had to explicitly aim at measuring the extent to which patients are able to take part in the crucial phase of their healthcare journey. We have listed and described in Table 4 the main instruments retrieved in the narrative review to measure patient engagement. In the table we also report the analysis of advantages and limitations of each measures provided by the experts who participated in the workshop dedicated to Query 2 and finally discussed, revised and agreed by the experts’ panel.

#### 3.2.2. Consensus Statement

The integrated assessment of Engagement, as an inspiring principle and value within the organizational and social healthcare system, is to be considered the *primo movens* of its promotion. Its assessment requires a monitoring across time at different levels of the healthcare system (i.e., nano-micro-meso-macro). Furthermore, the assessment of Engagement should not be addressed only to the “receivers of care”, but also to their families, and even to the healthcare professionals.

Thus, assessing the level of engagement of patients in the clinical care process should become a routine within clinical practice in order to evaluate the efficacy/efficiency of the clinical interventions and personalize them according to the characteristics of the recipients and to the complexity of their clinical care cases. Furthermore, the adoption of engagement assessment tools should be seen as a way to ensure that the voices of all the key stakeholders of a healthcare system are heard.

Just as important is the evaluation of healthcare professionals’ attitudes, beliefs and behaviors related to the promotion of the active role of the patient with chronic illness and his/her formal/informal caregivers along the care journey. Healthcare professionals’ skills/knowledge for the efficient promotion of Engagement need to be assessed and enhanced as well.

More attention should also be paid to the evaluation of family caregivers’ levels of engagement and to their needs in terms of psychological support and health literacy. Caregivers, as well as patients and healthcare professionals, should be listened to and requested to express their experiences, and priorities of engagement.

### 3.3. Query 3—What are the Best Practices to Promote Patient Engagement?

#### 3.3.1. Main Evidence

Both the systematic analysis of the literature and the *ad hoc* survey were devoted to collect evidence about good practices of patient engagement promotion. The literature reporting evidence on the effectiveness of such interventions which met our review criteria was quite limited (see Appendix B) thus testifying a debate still in its infancy [40]. The majority of interventions retrieved only addressed to the patient, with only a minority addressed to more than one stakeholder. This appears as a limitation of the current debate about patient engagement since engagement initiatives should include also the other stakeholders. Among the different strategies, motivational interviewing and goal setting [60,61], therapeutic education [62] and support to medical communication [63,64,65] were the most recurrent. On the other hand, collection of good practices with the stakeholders’ survey resulted in more variegated initiatives and tools, although with a lower level of scientific evidence. Furthermore, thanks to the survey and the experts’ workshop a great variety of tools was retrieved for each strategy, dedicated to the different stakeholders and not only to patients and caregivers (i.e., dedicated to support healthcare professionals, to support society and with a broader consideration of stakeholders to be involved in the intervention) and this compensated the relative limited number of evidence base practices retrieved in the literature and assessed with RCT (see Table 5).

#### 3.3.2. Consensus Statements

**Figure 2:**
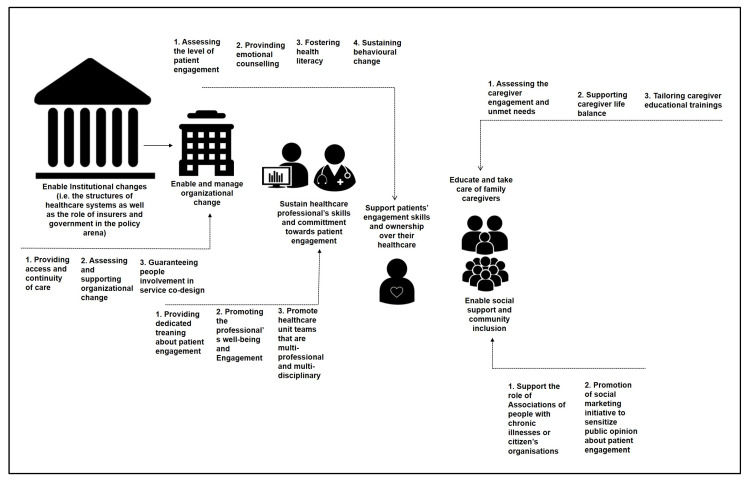
The engagement ecosystem.

The experts stated that it is necessary to avoid a simplistic approach to the engagement promotion, by limiting it to matters concerning just the individual patient. As stated above, the limited debate about the importance of developing multi-stakeholders initiatives to promote engagement is a current limitation according to the experts involved in this consensus conference process. Moreover, engagement is not simply a ‘tool’ to be incorporated into chronic care management; it is rather a fundamental and primary component of clinical work and a key skill for healthcare providers. Furthermore, initiatives should not be targeted only to the patients but they should also enable the other crucial actors (i.e., family caregivers, community members, healthcare professionals, policy makers…) to be sensitized, literate and behaviorally synergic with the principle of engagement promotion. Therefore, the consensus conference recommends developing an “ecosystem” of actions of engagement promotion on different levels: individual, interpersonal, organizational, social-community, and political/institutional, whose main goals are schematized in Figure 2. As in a real ecosystem, the goal is not only to better coordinate initiatives and actions at different levels of complexity of an healthcare system towards achieving the goal of engagement; but is also to foster a deep cultural change of each system component (i.e., patients, family, community, healthcare professionals, managers and organizations) to understand how much all their actions are interrelated and interdependent to really promote patient engagement. This because, as previously discussed, engagement is not only a duty of the patients but it is a care paradigm shift in all the components of the healthcare system planning, managing and delivering.

#### 3.3.3. More in Details

(1) At the level of patients, it is necessary to support their engagement skills and their sense of ownership over the healthcare course. Interventions have to be designed and structured on the basis of a scientific framework of engagement and personalized in accordance with the measurement of patients’ needs and willingness to take ownership of their health management. To achieve this goal, the following are recommended: a) Therapeutic education (i.e., interventions aimed at improving patient treatment by giving patients independence, and helping them to obtain and maintain the necessary skills to live more comfortably with their disease) and peer education (i.e., teaching and sharing health information, values and behavior in educating others who may share similar social backgrounds or life experiences); b) Motivational strategies and counselling; c) Implementing psychology counselling interventions to increase motivation and self-awareness.

(2) The participation of healthcare professionals in this cultural shift and support their knowledge and skill acquisition for Engagement promotion is crucial in order to avoid psychological resistances and to have them “on board”. To sensitize, train and involve healthcare professionals and the healthcare unit team, it is necessary to promote an “engagement culture” through concrete actions such as: a) Providing, starting from the university health professionals’ educational curricula with continuous training, knowledge and specific skills for the promotion of engagement in everyday clinical practice; b) Promote the health professional’s well-being and engagement and motivate him/her towards initiatives of engagement promotion; c) Promote healthcare unit teams that are multi-professional and where multi-disciplinarity is a prerequisite according to both literature and experts [66]. The joining dialogue and effort among different knowledge and expertise, where also patients experts can take a role, is an important factor which can foster the establishment of a real culture of engagement in healthcare organization, further then guaranteeing a better clinical effectiveness.

(3) Promoting engagement of family members and informal caregivers, through specific actions of education, sensitization and involvement, is an important resource to strengthen the efficacy of the preventive or therapeutic intervention. Focusing only on the people with illness is reductive and imprecise, as more often than not the person with chronic illness is not alone in his/her encounter with healthcare professionals, nor in taking charge of the management of his/her health. When the caregiver becomes an active part of the healthcare team and gains knowledge regarding the value of his/her role, s/he contributes to increase of the potential of therapeutic success of the interventions in both short and long term. In relation to informal/family caregivers, there is a need for: a) resources that guarantee efficacy, sustainability and continuity of the interventions in the long run; b) emotional support and counselling; c) caregiver education and improvement of health literacy.

(4) Models, processes, and practices of the social and healthcare organization should be reconfigured in order truly to achieve the goal of engagement. Opting for a personalized care intervention and a better continuity of social welfare services are conceived as fundamental precursors of patient engagement. Institutional changes at the level of the National Healthcare System are crucial in order to enable the promotion of a true engagement ecosystem. At the same time the role of policy making is needed to be consistent with the goals of achieving engagement. In this regard, the following priorities are recommended: a) Integration of assistive, social and care services; b) the establishment of case managers; c) the continuity and personalization of social welfare and care interventions, also enabled by institutional changes at the level of the National Healthcare System; d) systematic stakeholders’ involvement in the co-design and co-production of care services and healthcare policies;

(5) Lay associations involving people with chronic illness, caregivers and volunteers can act as a ‘glue’ of the different functions and organizations that are responsible for the management of the person with chronic illness. They are a priceless source of education, information, practical and especially emotional support for the assisted and their families. The valuing and support of the role of associations of people with chronic illnesses or citizen’s organizations within the “eco-system” for the promotion of engagement benefit the assisted, his informal/family caregiver and the social and healthcare system itself, both in terms of protection of their rights and of promotion of the knowledge and exercise of the duties of the people thanks to their irreplaceable contribution in the realization of their health plan.

(6) Finally, the promotion of initiatives of social and public opinion information and sensitization about the value of engagement is to be considered. Society, in particular peer networks, can have a crucial role in the promotion of engagement for the patient and his/her informal caregiver. Social sensitization and informative initiatives about the value of engagement as well as about a transparent analysis of the challenges encountered by patients in the engagement process are desirable. Sensitization and informative campaigns, with a social marketing approach, can be useful in the achievement of this goal.

### 3.4. Query 4—What is the Role of the New Technologies in the Promotion of Patient Engagement?

#### 3.4.1. Main Evidence

There is a broad agreement in the scientific literature and among experts that technology can be considered a facilitator of Engagement and could be integrated with other types of strategies. However, despite the fact that the number of published articles reporting on digital interventions to promote engagement is increasing, in our systematic review of the literature only 47 articles were included in the final analysis because they met the expected quality criteria. Indeed, the randomized control trials conducted to assess the effectiveness of digital health interventions to promote engagement are still a minority: many interventions are still in their infancy and not yet verified in their effectiveness. Moreover, not many digital health interventions are expressly tested to improve engagement and measured with reliable and scientific validated measurements.

However, although scientific evidence about the effectiveness of digital technologies in promoting engagement is still in its infancy, the experts interviewed agreed on the great potential of these tools in making the promise of patient engagement achievable. However, they also raised some concerns in regard to the process of technologies design and implementation in real world healthcare settings.

#### 3.4.2. Consensus Statement

Technological interventions for the promotion of engagement should be perceived as integrating and complementary, and not substituting the traditional strategies and interventions for the education and support of the patient. The efficacy of technological intervention is in function of the assistive and cure relationship. Moreover, it is necessary to avoid the risk that these are developed and used exclusively as something “imposed from above” (i.e., the welfare system) on people with chronic illnesses. It is important to involve the final users (people with chronic illness and their caregivers) in the design and implementation of the technology so that it can respond to the specific needs of the different phases of engagement. Furthermore, the design and implementation of technological initiatives for the promotion of Engagement should be defined in collaboration with the healthcare system and its professionals in order to ensure the best possible alignment of healthcare models and practices with the information systems and procedures currently in use.

Technologies should be designed as customizable according to the specific level of engagement previously measured with adequate and scientific instruments, derived from an analysis of the engagement needs of the different stakeholders involved in the process, to improve the personalization of the technological intervention.

The healthcare system must, on its side, promote the definition of policies, principles and criteria aimed at regulating the design and implementation of initiatives for the promotion of engagement that are able to safeguard people’s health. However, efficacy assessment, feasibility, as well as regulatory mechanisms and certification criteria must be provided for an appropriate use of these instruments.

## 4. Discussion

Engagement-promoting strategies vary according to clinical conditions, socio-demographics characteristics of the patients and seriousness of their symptoms: it is important to adopt a systemic approach that takes account all of the obstacles that may be present at different levels. A systemic view of engagement (i.e., with the broad consideration and coordination of all the different levels of the healthcare ecosystem) is needed in order to promote coordinated and integrated actions aimed at promoting and sustaining it. Engagement should be seen as a multi-stakeholders process. Furthermore, the adoption of instruments for the assessment of the level of engagement of a person with chronic illness and/or his/her informal caregiver, is a crucial strategy to overcome the “one size fits for all” logic and instead move in the direction of modular actions that can be fine-tuned throughout the person’s clinical care journey. Finally, the “patient” is not the only actor that needs to be considered and supported to achieve the engagement goal; on the contrary an eco-system of actions should be enacted at the different levels (i.e., nano-micro-meso-macro) and addressed to the various stakeholders (i.e., patients, family caregivers, healthcare professionals, society, organizations and peer networks). Furthermore, engagement-oriented policy making and institutional changes at the level of the National Health System should be foreseen in order to enable the real application and realization of an engagement ecosystem.

## 5. Conclusions

This consensus conference was established in response to concerns about the rapid increase in the number of patient engagement initiatives being developed around the world. The consensus Conference prioritized the need for a set of principles to guide the quality appraisal of patient engagement interventions (i.e., synthesis of the main recommendations emerged in Table 6 and in Figure 2). The purpose was to enhance the quality and effectiveness of patient engagement strategies, establishing a shared evidence framework for the contents, development, implementation and evaluation of them. Particularly, the claim of achieving and engagement ecosystem aims at being a compass for less fragmented initiatives of patient engagement in favor of better organized and coordinated actions which take into consideration the different levels (micro-meso-macro and mega) of the healthcare systems.

The *Consensus Conference on Patient Engagement* is an inter-professional and cross-disciplinary endeavor engaging healthcare practitioners, researchers, policy leaders, patients, and caregivers in the development of recommendations for transfer of patient engagement into real world practice. Significantly, it goes beyond the mere endorsement of Engagement to the translation of this principle into action through use of validated tools and strategies to be applied in both educational interventions and clinical practice. Furthermore, the consensus conference process has to be envisaged as an engagement in the research process itself: patients as well as their unformal caregivers were deeply involved throughout the entire process of evidence collection, discussion and prioritization. They were not considered an “object” of discussion but as “subject” claimed to speak their voice in a continuous process of negotiation with the other key stakeholders of the healthcare arena: healthcare professionals, scientists and policy makers. Thus, this consensus conference is itself a process of engagement in order to set standards and criteria for a less tokenistic debate about the topic and to ensure more effective transferability of research insights into real world clinical practice. The shift towards a real culture of Engagement at all the levels of the healthcare system will be the base for a real implementation of new actions able to magnify the starring role of patients and their caregivers along the whole care journey.

### Limitations

While the process to develop the consensus statement was comprehensive, thorough, and inclusive, there were some important limitations. First, its focus was only on chronic care. Although the consensus statement acknowledges that patient engagement is crucial for every patient involved in a care process, the consensus conference process was more focused on the engagement of patient with chronic conditions. Moving forward, the definition should be periodically revisited, and if necessary updated, to ensure it conveys patient engagement as a priority for healthcare, and perhaps to determine if and what changes are required for acute care or prevention settings. For this reason, these recommendations should be tested in other settings.

Second, generalizations from the literature scan and expert workshops must be made with caution because they reflect the expertise and opinions of the experts involved in the process and a future replication of the consensus process in a broader international scope would be desirable in order to incorporate also the experiences and advices of other experts on the topic. Furthermore, recommendations related to tools and initiatives for promoting patient engagement may be limited due to the main focus on RCT studies on patient engagement of the systematic review of the literature conducted to answer Query 3 and 4. The limitedness of this selection criteria has been balanced by including the experts’ perspective in the whole process.

As mentioned above, another limitation is that this consensus statement reflects the opinion of approximately 100 individuals, who participated in the process either as workshop experts or as contributors to the stakeholders’ survey. It may be that another set of participants might have come to slightly different conclusions. However, this critique is not unique to our consensus paper. Critical issues are the selection of experts and of the study leaders, the design and method of the consensus forming process like the use of questionnaires, the definition of a sufficient level of agreement. However, considering the wide variety of participants involved, with expertise in different fields of clinical medicine, and in the psychosocial domain, the results are likely to reflect the requirements of medical practice. Furthermore, the methodological rigor of the process has been assessed and guaranteed by expert methodologists with the role of supervising each methodological steps of the consensus conference, and this in order to minimize every source of distortion. Furthermore, all experts involved signed a conflict of interest statement, in order to carefully assess eventual biases in the input and the feedbacks provided.

In addition, further analysis and discussion would be worthy to expand recommendations related to the prerequisite and advised actions to sustain engagement at the organizational and policy levels, apart from sustaining patient advocacy process and the analysis of organizational models, culture and metrics to assess the engagement ecosystem functioning as a whole. This point is worthy to be explored in a future cross-country validation of the recommendations drafted.

Finally, these recommendations should not be conceived as strict guidelines to practice. Rather they are a scientific systematization of best practices often fragmented and parceled in the medical sector. These recommendations should be envisaged as inspirational principles to promote a real eco-system of engagement in chronic care, against a reductive and unrealistic approach to this issue.

Another limitation is linked to the local nature of the consensus conference process. The “requirements of medical practice” may be different in different nations and communities. However, although this project took place in Italy, the experts involved have a renowned international scientific and clinical expertise on the topic of the conference and they developed their recommendation according to international scientific evidences. However, a further cross-national validation of the recommendation provided by this consensus statement is warranted and is currently ongoing. Furthermore, additional analysis and discussion should be provided in relation to the applicability of such recommendation in the different healthcare systems which have different organizational structure and models across countries. For instance, this consensus was reached with specific reference to the Italian national healthcare system and its public structure, while in a private healthcare system, like the American one, further consideration should be done relating to other actors such as health insurers and private healthcare organizations.

## Figures and Tables

**Table 1:** Methodological process to conduct the literature analysis.

Analysis of the Scientific Evidence Related to Queries 1 and 2	
*Method and Search Strategy*	For queries 1 and 2 the literature analysis followed the principles of conceptual analysis, widely spread in social science research [8,37,38]. Cochrane Library, Isi Web of Science, PubMed, Scopus, CINAHL, PsychInfo were subject to a systematic search according to the following keywords search string: [“patient engag*” OR “consumer engag*” OR “client engag*” OR “citizen engag*”] AND [“definition” OR “conceptualization” OR measure OR “questionnaire”]. No restriction was applied regarding the year, the language or the document type. Additional key references were added and analyzed on the basis of their inclusion in the bibliographical references lists of the studies initially selected for the analysis.
*Selection Process*	In the analysis, only the manuscripts that reported a conceptual definition or a modelling theory of the concept of patient engagement were included. The manuscripts were considered as “conceptual” if they discussed in depth the theoretical underpinning of a construct and its determinants and characteristics. Furthermore, careful attention was given to the modality of operationalization and measurement of the theoretical constructs proposed in the analyzed studies. First, the duplicates generated from the systematic search were delated. At a later stage, all the titles and abstracts found were read and analyzed with the aim of excluding irrelevant and incoherent sources with the study inclusion criteria. Finally, the full texts were read and thoroughly analyzed to understand how they conceptualized, described and operationalized the concept of patient engagement. The process of analysis of the sources was ongoing until no more meaning references were retrieved.
**Analysis of the Scientific Evidence Related to Queries 3 and 4:**	
*Method and Search Strategy*	With the aim of answering queries 3 and 4, Cochrane Library, ISI Web of Science, PubMed, Scopus, CINAHL, PsychInfo were the object of a systematic research conducted with the following search string: (“patient* engag*”) AND [“plan*” OR “practice*” OR “intervention*” OR “program*” OR “protocol*” OR “trial*”]. No restriction was applied regarding the year, the language or the type of document. The search was integrated on the basis of an accurate analysis of the bibliographical references reported in the studies found.
*Selection Process*	We adopted the following inclusion criteria: (1) Years covered by the research: all the literature produced until the year 2016; (2) Population: studies that explicitly discussed the concept of Patient Engagement in the context of chronic illnesses; (3) Types of studies: with the aim of focusing the analysis on the most significant scientific evidence, only studies with Randomized Controlled Trial were included. Although we are aware that due to the infancy of the scientific debate about patient engagement the number of RCT on programs aimed at promoting patient engagement might be in a limited number, we preferred to keep this restrictive criterion in order to assess the maximum level of evidence achieved in the scientific literature about patient engagement initiatives. The limitedness of this decision has been however complemented by the following steps of the process: i.e., the experts survey and the workshops. The identified studies underwent another selection through the analysis of the titles and abstracts, to which followed an exclusion of those that were clearly unsuitable for the queries and inclusion criteria previously described. Of all the selected abstracts the full texts were then obtained and divided per topic area (in reference to the CC queries) and types of study. The systematic search of the sources was purposefully initially broad, in order to include all of the potentially relevant studies for the study objectives. The articles found were then further selected in a second phase of screening. Specifically, during an initial selection phase all the sources found were analyzed with regard to their title and abstract. This analysis allowed for the selection of only the relevant studies according to the following criteria: (1) being a Randomized Controlled Trial; (2) referring to chronic patients; (3) presenting measurement data of the impact of the intervention finalized to increase Patient Engagement; (4) being a peer-reviewed article with full text availability.
Supplementary Materials (Tables S1 and S2) **Related to the Two Reviews Outcomes**	
*Main Outcome*	The results achieved by these two literature review were deeply commented and reported in two reports provided to the experts participating in the workshops and experts’ panel. Original reports are partially published [39]. The systematic literature review was partially published in this manuscript [40]

**Table 2:** Distributions of experts by disciplines.

Discipline	*N*	%
Medicine and biology	35	34
Psychology	24	23
Sociology	3	3
Nursing	15	14
Patient advocacy	11	10
Policy making	8	8
Public health	4	4
Health economics	3	3
Health engineering	1	1
Total	104	100%

**Table 3:** Engagement definitions selected from the narrative review performed to answer questions 1 and 2.

Author(s), Year [Reference]	Definition of “Engagement”	Setting of Application	Level of Analysis
Mahmud, 2004	It is a process of healthcare priorities definition. It consists in empowering people to provide input to decisions that affect their lives and encourages support for those decisions, which in turn improves the public’s trust and confidence in the healthcare system.	Health service design	Macro (organizational factors)
Dearing et al., 2005	Developing “engagement” means fostering those client-therapist working alliances that help the client to gain a more realistic understanding of the nature, process, and expected outcomes of treatment.	Healthcare communication/relationship	Micro level (relational factors)
Davis et al., 2007	Option for patients to be informed partners in their care, including a recasting of the care relationship where the clinician enacts the role of adviser, and patients or designated surrogates for incapacitated patients serve as the locus of decision making.	Healthcare communication/relationship	Micro level (relational factors)
Mc Bride et al., 2007	It is a process that allows, at different levels, the wider community to have a say in the future direction of people’s health care.	Health policy design	Macro level (health policy factors)
Dunston et al., 2009	Dialogic and co-productive partnership among the healthcare system, healthcare professionals and citizen/healthcare consumers whereby these actors become co-productive.	Health service design	Meso level (organizational factors)
Forbat et al., 2009	[to engage patients means] working in partnership with service-users, keeping them informed about: (i) service redesign/improvement processes, (ii) policy, (iii) research and (iv) their own care/treatment. It also implies balancing powers between patients and health providers.	Health service design	Meso level (organizational factors)
Schley et al., 2011	Engaging clients in the therapeutic encounter means developing collaboration, perceived usefulness, and positive client/therapist interaction.	Healthcare communication/relationship	Micro level (relational factor)
Mulley et al., 2012	A process of shared decision making, described as a sequence of three types of conversation - team talk, option talk and decision talk. [engaging patients] - means creating a preference diagnosis which has a unique profile of risks, benefits and side effects.	Healthcare communication	Micro level (relational factors)
Sanders et al., 2012	A collaborative, bidirectional process whereby patients’ knowledge and experience is shared in a dialogue with program developers, health practitioners and researchers. It involves actively harnessing the consumer’s voice to strengthen the quality, relevance and effectiveness of an intervention.	Healthcare design	Meso level (organizational factors)
Carman et al., 2013	Patients, families, their representatives, and health professionals working in active partnership at various levels across the health care system—direct care, organizational design and governance, and policy making—to improve health and health care.	Healthcare deign	Macro level (policy factors)
Patel et al., 2013	The [engaged] patients have the ability to balance clinical information and professional advice with their own needs and preferences. It is a collaborative approach where shared decision making, equal distribution of power and exchange of clinical information are enacted.	Healthcare communication	Micro level (relational factors)

**Table 4:** Instruments for engagement assessment.

Scale Name	Characteristics	Pros	Cons	Recommended Use to
**Patient Engagement Assessment Scales**				
***Altarum Consumer Engagement (ACE)* [49]**	15 item scale that assesses the individual’s behavior in managing his/her health and his/her decision-making regarding heath care. The instrument consists of four sub-scales (commitment, informed choice, navigation, ownership) each indicative of a specific aspect of Engagement.	The scale is very detailed and allows for good assessment of the patient’s self-management skill.	The scale is quite long and complicated in its clinical application. The scale does not measure the emotional-motivational component of Engagement.	Assess cognitive and behavioural attitudes of patients in self-management
***Patient Activation Measure*** **(PAM) [43]**	Scale formed of 13 items that assess the person’s current behavioural abilities in managing the illness and the treatment prescriptions.	The scale is broadly used and has validation in several languages	It focuses on the behavioural and cognitive components of Engagement, and does not analyze the emotional-motivational component of Engagement	Assess cognitive and behavioural attitudes of patients in self-management
***Patient Health Engagement Scale (PHE-s)* [50]**	5 item scale developed on the basis of a solid conceptual evidence-based model of the patient with chronic illness’ experience of Engagement (PHE-model). The scale assesses the ability to reconfigure ones’ identity from passive receiver to co-author of the heath service.	The scale is easy and fast to use in the clinical context. The scale is very reliable in measuring the psychological attitude of patients towards Engagement	The scale does not measure behavioural components of the patient’s self-management.	Assess emotional and motivation readiness of patients to engage in the healthcare journey
***The Partners in Health (PHI) Scale*** [51]	Developed by Battersby and colleagues (2003), the PIH is a generic assessment scale for patients managing their chronic medical conditions. The scale consists of 11 items aimed at measuring the patients’ features regarding the following dimensions: (1) Knowledge of the condition and various treatment options; (2) Ability to negotiate a plan of care; (3) Engagement in activities that protect and promote health; (4) Monitoring and management of the symptoms and signs of the clinical condition(s). Both patients and health professionals judged the scale as acceptable and easy to use, and the health professionals endorsed its clinical utility	The scale enables a multidimensional analysis of engagement in the various domains of patients’ experience. It also allows to mirror patients’ and healthcare providers’ evaluations of engagement	The scale cannot be used to measure the emotional and motivational components of the engagement experience. The scale is quite long and complex to used	The scale is usable for an initial and very comprehensive assessment of engagement, but it is less usable in reiterative assessment due to the time of completion
***The Patient Enablement Instrument (PEI)* [52]**	The Patient Enablement Instrument (PEI) – developed by Howie and colleagues (1998) is designed to measure the patients’ ability to understand the nature of their problems and cope with their illness. This tool addresses six questions regarding a patient’s recent consultations, and, assess the following areas: how much they felt able to (1) cope with life, (2) understand their illness, (3) cope with their illness, (4) keep healthy, (5) feel confident about their health, and (6) able to help themselves. Scores were categorized to indicate low (0–4), medium (score 5–9), and high enablement (10–12)	The scale has been widely used and found to be reliable (Cronbach’s α = 0.897).	The scale is mainly related to patients’ ability to understand the nature of their problem and the way to cope with it and it is only related to the consultation experience	The PEI was developed to be used in primary care and is to be completed by the patient after consultation.
***The Self-Management Ability Scale (SMAS)*** [53]	Developed by Cramm and colleagues (2012) and based on the self-management of well-being (SMW) theory (Steverink et al., 2005), the 30-item Self-Management Ability Scale (SMAS) measures self-management abilities (SMA). The 30-item SMAS consists of six five-item subscales. Each sub-scale assesses one of the six core abilities to form the composite construct of self-management: (1) take initiatives (be instrumental or self-motivating in realizing aspects of well-being); (2) invest in resources for long-term benefits; (3) maintain variety in resources (achieve and maintain various resources for each dimension of well-being); (4) ensure resource multi-functionality (gain and maintain resources or activities that serve multiple dimensions of well-being simultaneously and in a mutually reinforcing way); (5) self-efficaciously manage resources (gain and maintain a belief in personal competence to achieve well-being); and (6) maintain a positive frame of mind. Each of these abilities directly relates to the dimensions of well-being specified in the SPF theory: physical well-being (comfort and stimulation) and social well-being (affection, behavioral confirmation, and status)	The scale is a very comprehensive tool to explore patients’ attitudes, beliefs, competences and self-efficacy perceptions when managing their health and treatment	The scale is quite long and complex, although articulated into several sub-scales. Furthermore. the scale is only related to engagement in self-management	The SMAS is usable to assess patients’ skills and attitude in self-management
***Self-Efficacy for Managing Chronic Disease Scale (SEMCD)*** [54]	The SEMCD scale was developed to assess outcomes of the Stanford Chronic Disease Self-Management Program (CDSMP), which aimed to enhance patients’ self-efficacy for chronic disease self-management. The current measure is built from six of ten items from the original Self-efficacy to Manage Symptoms and the Self-efficacy to Manage Disease scales (2 of 10 scales designed to evaluate the Stanford program).	German, Persian, and an international validation study of both the English and Spanish versions all show the SEMCD to be uni-dimensional and internally consistent	Despite extensive use, little information is available on how the original scales were designed or items selected.	The scale is usable to assess patients’ behavioural ability to manage the disease and their self-effectiveness in doing it.
***Hopkins Rehabilitation Engagement Rating Scale (HRERS)*** [55]	Hopkins Rehabilitation Engagement Rating Scale (HRERS) provides an evaluation of the degree of patients’ participation in the rehabilitation process. It consists of a 5-item measurement with which to assess clinicians’ evaluation of patients’ engagement in acute rehabilitation services	The scale is widely used in rehabilitation settings and enables clinicians to rate their patients’ level of participation on the basis of agreed standards	The measurement is only concerned with the rehabilitation setting and only provides a clinician rating of patient engagement	The scale was developed for the acute rehabilitation setting
**Family and Caregiver Engagement Assessment Scales**				
***Preparedness for Caregiving Scale*** **[56]**	An 8-item questionnaire (validated on a psychometric level) created to assess the extent to which the caregiver perceives him/herself as prepared to deal with the assistive role on various levels (physical care, emotional support, stress management).	The scale furnishes an adequate measurement of the caregiver’s competences in care-taking as well as his/her psychological adjustment to assuming this role.	The scale was developed and validated for the neurological and oncological field and has never been used in other therapeutic areas.	The scale is devoted to assessing caregivers’ experience in neurological and oncological settings
***Parent-Patient Activation Measure* [57]**	This is the caregiver version of the 13-item scale that measures the patient’s activation in self-managing his/her healthcare. This scale assesses the level of the caregiver’s activation, evaluating his/her knowledge, his/her perceived self-efficacy and his/her desire to take an active role in managing the healthcare of his/her loved one.	The scale is similar to the one aimed at measuring patient activation and allows to mirror patients’ and caregivers’ experiences	The scale is specific for the pediatric area.	The scale is the analogue of PAM version for patients and it is dedicated to parents of pediatric patients
**Scales for the Assessment of Healthcare Professionals’ Attitudes towards the Principles of Patient & Caregiver Engagement**				
***Clinician Support for Patient Activation Measure – CS-PAM* [58]**	Instrument that allows assessment of the attitudes and beliefs of clinicians towards the activation of the person with chronic illness.	This measurement is similar to the ones aimed at measuring caregivers’ and patient’s activation and allows comparisons and mirroring of the activation level among these actors.	The scale does not assess actual clinicians’ behaviors.	The scale is the analogue of the PAM version for patients and it measures healthcare professionals’ attitudes and beliefs about patients’ activation
***Self-Management Support (SMS) Scale* [59]**	Behavioural scale intended to measure the extent to which clinicians use strategies to improve the patient’s self-management competences.	Offers a list of prototypical behaviors that characterize the clinician’s abilities to motivate and educate the person in self-management.	It does not assess the clinician’s attitudes and his/her value orientation to Engagement.	The scale is useful to rate the number of HP behaviors dedicated to improve patients’ engagement

**Table 5:** Best practices from literature review and expert voices: a qualitative taxonomy.

Target	Patient Engagement Strategies	Examples of Methods and Tools Suggested by Experts
**Patient and Family**	Give information about the different aspects of the care	*Leaflets, books, multimedia platforms, learning video, websites, seminars/workshop/conferences*
	Motivate in improving their awareness about their functioning, their needs and their ability to change	*Motivational interviews, goal setting, problem solving techniques, wellness plans, behavioural counselling*
	Improve the self-awareness	*Mindfulness interventions, narrrative diaries, expressive writing*
	Support the psychological and emotional elaboration	*Psychological consultations, self-help groups/patients’ groups, positive psychology*
	Communicate with the clinicians	*Question asking, decision aids, teach back methods*
	Self-monitor	*Therapies patients’ diaries*
	Protect patients’ rights	*Patient advocacy group, informed consent*
	Promote the networking	*Patient associations, patient voluntary associations, caregivers associations*
**Health Professionals**	Sensitize clinicians to the value of patient engagement	*Scientific literature sharing and discussion, seminars, workshops, conferences, continuing and distance education*
	Train clinicians to communicate more effectively with their patient	*Clinical cases, role playing, consultations’ simulations*
	Support to the professional identity and role	*Leadership trainings, professional identity consultancy, professional counselling*
	Promote the clinicians’ wellbeing and work engagement	*Health promotion interventions, burnout assessment, work engagement interventions, psychological consultancy*
**Healthcare Organization**	Foster a multidisciplinary approach to the care	*Multidisciplinary equipe, interdisciplinary team meetings*
	Facilitate the continuity of care as an essential part of enabling better health outcomes, through the boost of patient engagement	*Case managers, personalized care plans, integrated care models, Electronic Health Records, Personalized Health Records, Bedsides shift reports*
	Measure the performance and the level of patient engagement	*Patient engagement measure tools (PAM, PHE-s, Altarum Consumer Engagement Scale), patient satisfaction measure, healthcare costs monitoring, public reports, accountability*
	Promote the active participation of all the healthcare stakeholders (i.e., patients and family organizations, clinical societies, policy makers….)	*Action research, groups of participative governance, focus group*

**Table 6:** Reports of the recommendation in synthesis.

Summary of Main Consensus Statements and Recommendations
“Engagement” in the clinical care field of chronicity is an umbrella concept that includes and extends beyond other concepts such as *adherence, compliance, empowerment, activation, health literacy, shared decision making*. Engagement is a complex process that arises from the combination of different dimensions and individual, relational, organizational, social, economic and political factors that connote the quality of life of the patient
The formal/informal caregiver - especially in the case of elderly people or young children with severe disabilities and/or in clinical conditions that make them less autonomous in their health management - plays a key role in the process of engagement
The evaluation and measurement of the engagement of all actors involved in the care process (patients with chronic illnesses, caregivers and healthcare professionals in the social field) is a factor crucial for enhancing the effectiveness and efficiency of the clinical care interventions
The training and sensitization of health professionals and healthcare organizations and policy makers about the principles of engagement
Technologies can enable the process of engagement and supplement other non-technological intervention strategies; but they are not an alternative to the therapeutic relationship
The possible results expected from engagement are: facilitation of the patient in taking care of his/her health, improvement of clinical results, improvement of lifestyle and reduction of healthcare costs, greater integration and continuity of the healthcare and social journey. However, the efficacy testing of these outcomes in the current literature is still quantitatively limited and methodologically weak: this may be due to the still “infancy” of academic research on the topic but also by the lack of investment on this crucial sector. Further joint research activities are needed internationally in order to produce evidence on the outcomes of Engagement initiatives.
It is necessary to promote further quality research in the field of efficacy testing of the methodologies and the impact of engagement in health and social services and in clinical care practice

## Supplementary Materials

The following are available online at https://www.mdpi.com/1660-4601/17/11/4167/s1, Table S1: Glossary of the key concepts of participatory medicine and their conceptual relationship with engagement, Table S2: Glossary of the main approaches and methodologies for promoting engagement.

## Appendix A

**Table A1:** Responsibilities and members of the Italian Consensus Conference on Patient Engagement.

Role	Responsibilities	Members
***Organizing Committee***	Was responsible for: - defining the aims of the conference; - finding financers; - identifying members of the Technical-Scientific Committee (TSC); - writing the protocol with the TSC; - promoting the conference; - organizing the different phases of the program; - identifying members of the Panel Jury; - formulating, in agreement with the Technical-Scientific Committee, the queries for the Panel Jury; - giving directions and methodological support to the Experts for the preparation of the reports to present to the Panel Jury; - defining the distribution and measuring strategies of the impact of the recommendations produced.	G. Graffigna, Università Cattolica del Sacro Cuore, Milano; A.C. Bosio, Università Cattolica del Sacro Cuore, Milano; S. Barello, Università Cattolica del Sacro Cuore, Milano; G. Castelnuovo, Università Cattolica del Sacro Cuore, Milano; M. Corbo, Casa di Cura Privata del Policlinico, Milano; G. Riva, Università Cattolica del Sacro Cuore, Milano
***Technical-Scientific Committee***	Was composed of members with recognized experience and representativeness identified and invited by the Organizing Committee and was responsible for: - collaborating with the Organizing Committee for the writing of the Consensus Conference protocol; - formulating the queries for the Panel Jury, in accord with the Organizing Committee; - internally designating its Experts and the eventual work groups needed to prepare and present to the Jury the reports of the single queries during the conference; - providing the Experts and the work groups with the necessary methodological directions to write the assigned reports and make sure that a common method is used to analyse data and present it to the Jury.	E. Anessi Pessina, CERISMAS (Centro di ricerche e studi in management sanitario), Università Cattolica del Sacro Cuore, Milano; R. Bellantone, Università Cattolica del Sacro Cuore, Roma; R. Borgatti, IRCSS Istituto Eugenio Medea, Bosisio Parini, Lecco; A. Celano, APMAR (Associazione Persone con Malattie Reumatiche); A. Cicchetti, ALTEMS (Alta Scuola di Economia e Management dei Sistemi Sanitari), Università Cattolica del Sacro Cuore, Roma; F. Consorti, SIPeM (Società Italiana di Pedagogia Medica); L. Coppola, DG Welfare Regione Lombardia; R. D’Elia, Ministero della Salute, Direzione Generale della Prevenzione; D. D’Ugo, SICO (Società Italiana Chirurgia Oncologica), Università Cattolica del Sacro Cuore, Roma; F. De Lorenzo, European Cancer Patients Coalition, FAVO (Federazione Italiana Associazioni di Volontariato in Oncologia); F. Donatelli, Università degli Studi di Milano, Istituto Clinico Sant’Ambrogio Gruppo San Donato; A. Fauci, Istituto Superiore di Sanità; F. Giardina, CNOP (Consiglio Nazionale Ordine degli Psicologi); P. Iannone, Istituto Superiore di Sanità; D. Mannino, AMD (Associazione Medici Diabetologi); D. Mari, Fondazione IRCSS Ca’Grande - Ospedale Maggiore Policlinico, Università degli Studi di Milano; V. Mastrilli, Ministero della Salute, Direzione Generale della Prevenzione; P. Mocarelli, IRCCS Fondazione Don Gnocchi; E. Molinari, Università Cattolica del Sacro Cuore, Milano, Istituto Auxologico Italiano; F. Molteni, Villa Beretta - Presidio di Riabilitazione dell’Ospedale Valduce; A. Muratore, SICO (Società Italiana Chirurgia Oncologica); G. Muttillo, IPASVI (Infermieri Professionali, Assistenti Sanitari e Vigilatrici di Infanzia); G. Perseghin, SID (Società Italiana Diabetologia); G. Pintori, Inversa Onlus (associazione italiana per i pazienti affetti da idrosadenite suppurativa-acne inversa-); E. Previtali, AMICI Onlus (Associazione Nazionale per le Malattie Infiammatorie Croniche dell’Intestino); W. Ricciardi, Istituto Superiore di Sanità; E. Rizzato, Fondazione SPP (Scuola di Sanità Pubblica); E. Santoro, IRCCS - Istituto di Ricerche Farmacologiche “Mario Negri”; G. Spata, FNMCeO (Federazione Italiana Ordine dei Medici); M. Tessarollo, Residenze Anni Azzurri (Gruppo KOS); R. Valdagni, IRCCS Istituto Nazionale dei Tumori.
***Panel Jury***	Had the authority to: - write a regulation of discussion in which the methods and procedures that the Jury will apply internally are defined a priori, including the composition of the Writing Committee; - read and evaluate the documents produced from the work groups; - assist to the presentation and discussion of the reports during the meetings of the Consensus Conference; - discuss, review and approve the preliminary consensus document to present at during closing time of the conference; - review and approve the final consensus document according to the modalities and times provided by the regulation.	A. Aglione, FAVO (Federazione Italiana Associazioni di Volontariato in Oncologia); G. Artioli, Arcispedale Santa Maria Nuova - IRCCS di Reggio Emilia; F. Avolio, Agenzia Regionale Sanitaria della Puglia; European Innovative Partnership for Active Healthy Ageing; C. Colombo, IRCSS Istituto di Ricerche Farmacologiche Mario Negri, Milano; S. Leone, A.M.I.C.I. Italia Onlus (Associazione Nazionale per le Malattie Infiammatorie Croniche dell’Intestino); M.C. Ghiotto, Regione del Veneto; B. Mazzoleni, Commissione Nazionale Ipasvi (Infermieri professionali, assistenti sanitari e vigilatrici di infanzia); R. Mete, Istituto Superiore di Studi Sanitari, Giuseppe Cannarella; P. Mosconi, IRCSS Istituto di Ricerche Farmacologiche Mario Negri, Milano; S. Nardi, Coordinamento nazionale delle Associazioni di Malati Cronici (CnAMC); C. Pinto, AIOM (Associazione Italiana Oncologia Medica); P. Quintaliani, SIN (Società Italiana Nefrologia), FIR (Fondazione Italiana Rene); G. Sanna, METIS FIMMG (Federazione italiana medici di medicina generale); S. Tonolo, ANMAR (Associazione Nazionale Malati Reumatici); A. Virzì, Società di Medicina Narrativa
**President of the Jury**	Had the authority to: - write the work regulation and get the Jury’s members to approve it; - verify that all the members of the Jury promptly receive the materials produced by the experts and work groups; - coordinate the Jury and the Writing Committee until the writing of the final consensus document; - regulate the unfolding of the Jury’s discussions, ascertain the poll results and countersign the meeting’s reports; - maintain the relationships with the Organization Committee and act as a conduit for communications directed to the Jury; - take part in the celebration of the Consensus Conference; - publicly communicate, at the end of the Jury’s discussion, the conclusions reported in the approved preliminary consensus document.	G. Damiani, Policlinico Universitario Agostino Gemelli, Università Cattolica del Sacro Cuore, Roma
***Writing Committee***	Formed by members selected from the Jury, this Commitee reflects the competences and characteristics of the Panel’s multidisciplinarity, provided the newsroom with the final consensus document, following the modalities established and described in the Jury regulation. This document is an integration of the preliminary document that the Jury will produce in the hours following the Consensus Conference with a synthesis of the tasks that the Panel based itself on to formulate the recommendations. Moreover, the Writing Committee verified the coherence between the conclusions and the accompanying texts.	F. Avolio, Agenzia Regionale Sanitaria della Puglia; European Innovative Partnership for Active Healthy Ageing; G. Artioli, IPASVI Emilia Romagna (Infermieri professionali, assistenti sanitari e vigilatrici di infanzia), Università degli studi di Parma; S. Leone, A.M.I.C.I. Italia Onlus (Associazione Nazionale per le Malattie Infiammatorie Croniche dell’Intestino); P. Mosconi, IRCSS Istituto di Ricerche Farmacologiche Mario Negri, Milano
***Scientific Secretariat***	coordinated the collected and exchanged material and information between the different participants involved	G. Graffigna, Università Cattolica del Sacro Cuore, Milano; S. Barello, Università Cattolica del Sacro Cuore, Milano
***Organizational Secretariat***	coordinated the operative organization of the conference	J. Menichetti, Università Cattolica del Sacro Cuore, Milano; M. Savarese, Università Cattolica del Sacro Cuore, Milano
***Members of the Expert Meetings***	Held the role of assessing and synthesizing the evidence present in literature that were pertinent to the queries of the Consensus Conference. Particularly, they had the following jobs: - preparing a synthesis of the scientific evidence available on the subject; - preparing a synthesis of the information available to the public that comes from different sources, regarding the subjects of interest in the conference; - handing the reports made to the Jury, within the stipulated times; - presenting the data collected during the celebration of the conference and participate in the discussion.	*Working Group on the definition of Patient Engagement:* A. Bertoni, Università Cattolica del Sacro Cuore, Milano; S. Donato, Università Cattolica del Sacro Cuore, Milano; S. Gilardi, Università degli studi di Milano; C. Guglielmetti, Università degli studi di Milano; M. Lastretti, Ordine degli Psicologi del Lazio; L. Lombi, Università Cattolica del Sacro Cuore, Milano; S. Ostuzzi, ALOMAR (Associazione Lombarda Malati Reumatici); G. Pitacco, ASUIT (Azienda Sanitaria Universitaria Integrata di Trieste); M. Savarese, Università Cattolica del Sacro Cuore, Milano; E. Vegni, Università degli Studi di Milano; N. Visalli, AMD (Associazione Medici Diabetologi) *Working group on the measurement of patient engagement*: S. Barello, Università Cattolica del Sacro Cuore, Milan; D. Bettega, Fatebenefratelli Ospedale Sacra Famiglia Erba (Como); F. Lucchi, Azienda Ospedaliera Spedali Civili Brescia; M. Magri, IPASVI Milano, Lodi, Monza e Brianza (Infermieri professionali, assistenti sanitari e vigilatrici di infanzia); M. Pozzi, Fatebenefratelli Ospedale Sacra Famiglia Erba (Como); L. Provenzi, IRCSS Istituto Eugenio Medea, Bosisio Parini, Lecco *Working group on the promotion of patient engagement:* M. Annoni, Fondazione Umberto Veronesi; M. P. Arnaboldi, IEO (Istituto Europeo Oncologico); L. Bellardita, IRCSS Istituto Nazionale Tumori; C. Carzaniga, GITIC (Gruppo Italiano Infermieri di Area Cardiovascolare); A. Castaldo IRCSS Istituto Piccolo Cottolengo Don Orione L. Garrino, SIPeM (Società Italiana di Pedagogia Medica); M. Gorli, Università Cattolica del Sacro Cuore, Milano; M. Gulizia, ANMCO (Associazione Nazionale Medici Cardiologi Ospedalieri) A. Lotti, SIPeM (Società Italiana di Pedagogia Medica); J. Menichetti, Università Cattolica del Sacro Cuore, Milano; M.L. Mottes, Diabete Forum; A.D.P.Mi Onlus (Associazione Diabetici della Provincia di Milano); N. Piana, Università degli Studi di Perugia; G. Quaglini, Parkinson Italia Onlus G. Scaratti, Università Cattolica del Sacro Cuore, Milano; M. Tettamanti, GITIC (Gruppo Italiano Infermieri di Area Cardiovascolare); P. Varese, FAVO (Federazione Italiana Associazioni di Volontariato in Oncologia). *Working group on the use of new technologies for patient engagement:* S. Bigi, Università Cattolica del Sacro Cuore, Milano; D. Bruttomesso, SID (Società Italiana di Diabetologia); L. Del Campo, FAVO (Federazione Italiana Associazioni di Volontariato in Oncologia); S. Franco, Istituto Superiore di Studi Sanitari, Giuseppe Cannarella; A. Mazzone, FADOI (Federazione delle Associazioni dei Medici Internisti Ospedalieri); G. Palumbo Villa Beretta - Presidio di Riabilitazione dell’Ospedale Valduce; D. Pero AIMAC (Associazione Italiana Malati di Cancro); G. Pintori, Inversa Onlus; G. Polvani, Università degli Studi di Milano, IRCSS Centro Cardiologico Monzino E. Santoro, IRCCS - Istituto di Ricerche Farmacologiche “Mario Negri”; S. Triberti, Università Cattolica del Sacro Cuore, Milano; A. Tzannis, Università Cattolica del Sacro Cuore, Milano

## Appendix B

**Table A2:** The main evidences from the systematic literature review in the answer to query 3 and 4.

Title	Authors	Year	Characteristics of the Intervention	Type of Intervention	Use of Technology
A smoking-cessation intervention for hospital patients	Stevens VJ, Glasgow RE, Hollis JF, et al.	1993	Multi-part inpatient intervention including counseling, videotapes, self-help materials and a phone call	Psychological support	yes
Patient empowerment and feedback did not decrease pain in seriously ill hospitalized adults	Desbiens NA, Wu AW, Yasui Y, Lynn J, Alzola C, Wenger NS, Connors AF Jr, Phillips RS, Fulkerson W.	1998	educational and empowerment nurse clinician-mediated intervention to relieve pain	Therapeutic education	no
Effects of preparatory videotapes on self-efficacy beliefs and recovery from coronary bypass surgery.	Mahler HI, Kulik JA.	1998	three types of videotapes	Psychological support	yes
A multicomponent intervention to prevent major bleeding complications in older patients receiving warfarin. A randomized, controlled trial	Beyth RJ, Quinn L, Landefeld CS	2000	multicomponent comprehensive program of warfarin management	Therapeutic education	no
The efficacy of playing a virtual reality game in modulating pain for children with acute burn injuries: a randomized controlled trial	Das DA, Grimmer KA, Sparnon AL, et al.	2005	virtual reality games	Therapeutic education	yes
Effect of education on blood pressure control in elderly persons a randomized controlled trial	Figar S, Galarza C, Petrlik E, Hornstein L, Rodríguez Loria G, Waisman G, Rada M, Soriano ER, de Quirós FG.	2006	self-management and patient empowerment workshops	Therapeutic education	no
A telephone-delivered empowerment intervention with patients diagnosed with heart failure	Shearer NB, Cisar N, Greenberg EA.	2007	telephone-delivered empowerment intervention to facilitate purposeful participation in goal attainment, self-management of disease, and perception of functional health	Psychological support	yes
Changes in diabetes distress related to participation in an internet based diabetes care management program and glycemic control	Fonda SJ, McMahon GT, Gomes HE, Hickson S, Conlin PR.	2009	Internet-based care management program	Therapeutic education	yes
Community based peer led diabetes self management. A randomized trial	Lorig, Kate, Ritter, Philip L., Villa, Frank J., Armas, Jean	2009	community-based, peer-led diabetes self-management program	Psychological support	no
Making the most of your healthcare intervention for older adults with multiple chronic illnesses	Hochhalter, Angela K. et al.	2010	patient engagement intervention for older adults with multiple chronic illnesses called Making the Most of Your Healthcare	Therapeutic education	no
Randomized control trial of the health empowerment intervention: feasibility and impact	Crawford Shearer NB, Fleury JD, Belyea M.	2010	Health Empowerment Intervention to promote the use of personal resources and social contextual resources	Psychological support	no
Multimedia education programme for patients with a stoma Effectiveness evaluation	Lo, Shu-Fen	2011	multimedia education programme	Therapeutic education	yes
Cluster-randomized trial of a mobile phone personalized behavioral intervention for blood glucose control	Quinn C. C., Shardell M. D., Terrin M. L., Barr E. A., Ballew S. H., Gruber-Baldini A. L.	2011	patient/provider web portals	Therapeutic education	yes
Efficacy of ongoing group-based diabetes self-management education for patients with type 2 diabetes mellitus A randomised controlled trial	Rygg, Lisbeth et al.	2012	diabetes self-management education	Therapeutic education	no
Enhanced medical rehabilitation increases therapy intensity and engagement and improves functional outcomes in postacute rehabilitation of older adults a randomized controlled trial	Lenze EJ, Host HH, Hildebrand MW, Morrow-Howell N, Carpenter B, Freedland KE, Baum CA, Dixon D, Doré P, Wendleton L, Binder EF.	2012	Enhanced Medical Rehabilitation	Therapeutic education	no
Effectiveness of peer-led self-management coaching for patients recently diagnosed with type 2 diabetes mellitus in primary care: a randomized controlled trial	Van der Wulp I, de Leeuw JR, Gorter KJ, Rutten GE.	2012	peer led self-management coaching programme	Psychological support	no
Effects of a Web-based intervention for adults with chronic conditions on patient activation: online randomized controlled trial	Solomon M., Wagner S. L., Goes J	2012	MyHealth Online, a patient portal featuring interactive health applications accessible via the Internet	Therapeutic education	yes
Effect of patient activation on self management in patients with heart failure	Shively, Martha J., Larson, Carolyn B.	2013	tailored face-to-face or telephonic program focused on having individualized self-selected health goals	Therapeutic education	yes
Effectiveness of a quality improvement intervention targeting cardiovascular risk factors: are patients responsive to information and encouragement by mail or post?	Senesael E, Borgermans L, Van De Vijver E, Devroey D.	2013	information and regular encouragement by email or letter on cardiovascular risk factors	Support to medical communication	no
Effectiveness of general practice based, practice nurse led telephone coaching on glycaemic control of type 2 diabetes the Patient Engagement and Coaching for Health PEACH pragmatic cluster randomised controlled trial	Blackberry ID, Furler JS, Best JD, Chondros P, Vale M, Walker C, Dunning T, Segal L, Dunbar J, Audehm R, Liew D, Young D.	2013	practice nurse led structured telephone coaching program	Therapeutic education	yes
Effects of a web-based patient activation intervention to overcome clinical inertia on blood pressure control cluster randomized controlled trial	Thiboutot J, Sciamanna CN, Falkner B, Kephart DK, Stuckey HL, Adelman AM, Curry WJ, Lehman EB.	2013	Web-Based Patient Activation Intervention with tailored messages suggesting questions to ask to improve blood pressure control	Support to medical communication	yes
Impact of single session motivational interviewing on clinical outcomes following periodontal maintenance therapy	Brand VS, Bray KK, MacNeill S, Catley D, Williams K.	2013	motivational interviewing	Therapeutic education	no
Internet based dyspnea self-management support for patients with chronic obstructive pulmonary disease	Nguyen HQ, Donesky D, Reinke LF, Wolpin S, Chyall L, Benditt JO, Paul SM, Carrieri-Kohlman V.	2013	Internet-based and face-to-face dyspnea selfmanagement programs	Therapeutic education	yes
The role of patient activation in improving blood pressure outcomes in Black patients receiving home care	Ryvicker, Miriam	2013	phone-based educational intervention for blood pressure skills building	Therapeutic education	yes
Online disease management of diabetes: engaging and motivating patients online with enhanced resources-diabetes (EMPOWER-D), a randomized controlled trial	Tang P. C., Overhage J. M., Chan A. S., Brown N. L., Aghighi B., Entwistle M. P., et al.	2013	(1) wirelessly uploaded home glucometer readings with graphical feedback; (2) comprehensive patient-specific diabetes summary status report; (3) nutrition and exercise logs; (4) insulin record; (5) online messaging with the patient’s health team; (6) nurse care manager and dietitian providing advice and medication management; and (7) personalized text and video educational ‘nuggets’ dispensed electronically by the care team	Therapeutic education	yes
Effect of a participant driven health education programme in primary care for people with hyperglycaemia detected by screening 3year results from the Ready to Act randomized controlled trial nested within the ADDITION Denmark study	Maindal HT, Carlsen AH, Lauritzen T, Sandbaek A, Simmons RK.	2014	Ready to Act programme with 2 individual counselling interviews and 8 group sessions for motivation, informed decision-making, action experience, social involvement	Therapeutic education	no
Effects of a patient oriented decision aid for prioritising treatment goals in diabetes Pragmatic randomised controlled trial	Denig, Petra et al.	2014	patient oriented decision aid	Support to medical communication	no
Reduction of inappropriate benzodiazepine prescriptions among older adults through direct patient education the EMPOWER cluster randomized trial	Tannenbaum C, Martin P, Tamblyn R, Benedetti A, Ahmed S.	2014	deprescribing patient empowerment intervention on benzodiazepine use and a stepwise tapering protocol	Therapeutic education	no
A salutogenic program to enhance sense of coherence and quality of life for older people in the community A feasibility randomized controlled trial and process evaluation	Tan, Khoon Kiat, Chan, Sally Wai-Chi, Wang, Wenru, Vehviläinen-Julkunen, Katri	2015	Resource Enhancement and Activation Program to develop the well-being and QoL of older people by strengthening their SOC, activation, and resilience through 24 activities	Psychological support	no
Effectiveness of motivational interviewing to improve therapeutic adherence in patients over 65 years old with chronic diseases A cluster randomized clinical trial in primary care	Moral RR, Torres LA, Ortega LP, Larumbe MC, Villalobos AR, García JA, Rejano JM; Collaborative Group ATEM-AP Study.	2015	motivational interviewing	Therapeutic education	no
Encounter Decision Aid vs Clinical Decision Support or Usual Care to Support Patient Centered Treatment Decisions in Osteoporosis The Osteoporosis Choice Randomized Trial II	LeBlanc A, Wang AT, Wyatt K, Branda ME, Shah ND, Van Houten H, Pencille L, Wermers R, Montori VM.	2015	encounter decision aid	Support to medical communication	no
Feasibility of Standardized Clinician Methodology for Patient Training on Hospital to Home Transitions	Wehbe-Janek H, Hochhalter AK, Castilla T, Jo C.	2015	standardized clinician methodology intervention	Therapeutic education	no
Patient Empowerment Improved Perioperative Quality of Care in Cancer Patients Aged ≥ 65 Years A Randomized Controlled Trial	Schmidt M, Eckardt R, Scholtz K, Neuner B, von Dossow-Hanfstingl V, Sehouli J, Stief CG, Wernecke KD, Spies CD; PERATECS Group.	2015	Patient empowerment intervention with information booklet and patient diary	Therapeutic education	no
Peer Coaches to Improve Diabetes Outcomes in Rural Alabama A Cluster Randomized Trial	Safford MM, Andreae S, Cherrington AL, Martin MY, Halanych J, Lewis M, Patel A, Johnson E, Clark D, Gamboa C, Richman JS.	2015	peer-coaching intervention	Psychological support	no
Safe and effective use of medicines for patients with type 2 diabetes A randomized controlled trial of two interventions delivered by local pharmacies	Kjeldsen LJ, Bjerrum L, Dam P, Larsen BO, Rossing C, Søndergaard B, Herborg H.	2015	individually targeted self-management intervention (1. brief, 2. extended)	Therapeutic education	no
Activating Patients With a Tailored Bone Density Test Results Letter and Educational Brochure: the PAADRN Randomized Controlled Trial.	Wolinsky FD, Lou Y, Edmonds SW, Hall SF, Jones MP, Wright NC, Saag KG, Cram P, Roblin DW; PAADRN Investigators..	2016	tailored patient activation letter communicating test results plus educational brochure	Therapeutic education	no
Assessing the effect of culturally specific audiovisual educational interventions on attaining self-management skills for chronic obstructive pulmonary disease in Mandarin- and Cantonese-speaking patients: a randomized controlled trial.	Poureslami I, Kwan S, Lam S, Khan NA, FitzGerald JM.	2016	culturally and linguistically specific audiovisual educational materials for COPD self-management	Therapeutic education	no
Cardiac rehabilitation using the Family-Centered Empowerment Model versus home-based cardiac rehabilitation in patients with myocardial infarction a randomised controlled trial	Vahedian-Azimi A, et al.	2016	hybrid cardiac rehabilitation programme with educational group sessions involving family members	Therapeutic education	no
Do empowered stroke patients perform better at self-management and functional recovery after a stroke? A randomized controlled trial.	Sit JW, Chair SY, Choi KC, Chan CW, Lee DT, Chan AW, Cheung JL, Tang SW, Chan PS, Taylor-Piliae RE.	2016	Health Empowerment Intervention for Stroke Self-management	Psychological support	no
Does a decision aid for prostate cancer affect different aspects of decisional regret, assessed with new regret scales? A randomized, controlled trial	van Tol-Geerdink, Julia J., Jan Willem H.	2016	Decision aid for radical PCa treatments	Support to medical communication	no
Effects of a home-based activation intervention on self-management adherence and readmission in rural heart failure patients: the PATCH randomized controlled trial.	Young L, Hertzog M, Barnason S.	2016	telephone-based patient activation care at home intervention	Therapeutic education	yes
Encouraging early discussion of life expectancy and end-of-life care: A randomised controlled trial of a nurse-led communication support program for patients and caregivers.	Walczak A, Butow PN, Tattersall MH, Davidson PM, Young J, Epstein RM, Costa DS, Clayton JM.	2016	nurse-led Communication Support Program for support end-of-life care	Support to medical communication	no
Increasing patient involvement in the diabetic foot pathway a pilot randomized controlled trial	McBride E, Hacking B, O’Carroll R, Young M, Jahr J, Borthwick C, Callander A, Berrada Z.	2016	Decision Navigation: a multicomponent intervention developed to promote informed treatment decision-making	Support to medical communication	no
Patient and Partner Feedback Reports to Improve Statin Medication Adherence: A Randomized Control Trial.	Reddy A, Huseman TL, Canamucio A, Marcus SC, Asch DA, Volpp K, Long JA.	2016	1) daily alarm and a weekly medication adherence feedback report; 2) plus sharing of alarm/report with a friend, family member, or a peer	Therapeutic education	yes
The effect of a patient centred care bundle intervention on pressure ulcer incidence (INTACT): A cluster randomised trial.	Chaboyer W, Bucknall T, Webster J, McInnes E, Gillespie BM, Banks M, Whitty JA, Thalib L, Roberts S, Tallott M, Cullum N, Wallis M.	2016	patient centred care bundle educational intervention ulcer incidence	Therapeutic education	no
The impact of sharing personalised clinical information with people with type 2 diabetes prior to their consultation A pilot randomised controlled trial	O’Donnell, M. et al.	2016	booklet including personalised clinical information	Support to medical communication	no
Effectiveness of personalised support for self management in primary care a cluster randomised controlled trial	Eikelenboom N, van Lieshout J, Jacobs A, Verhulst F, Lacroix J, van Halteren A, Klomp M, Smeele I, Wensing M.	2016	Patient feedback scale and personalized self-management support	Psychological support	no

## References

[1] Couto J.E., Comer D.M. (2012). Patient engagement: The critical catalyst to health reform in the USA. J. Comp. Eff. Res..

[2] Clancy C.M. (2011). Patient engagement in health care. Health Serv. Res..

[3] Coulter A. (2012). Patient engagement—What works?. J. Ambul. Care Manag..

[4] Gruman J., Rovner M.H., French M.E., Jeffress D., Sofaer S., Shaller D., Prager D.J. (2010). From patient education to patient engagement: Implications for the field of patient education. Patient Educ. Couns..

[5] Barello S., Triberti S., Graffigna G., Libreri C., Serino S., Hibbard J., Riva G. (2016). eHealth for patient engagement: A Systematic Review. Front. Psychol..

[6] Hibbard J.H., Greene J., Shi Y., Mittler J., Scanlon D. (2015). Taking the long view: How well do patient activation scores predict outcomes four years later?. Med. Care Res. Rev..

[7] Graffigna G., Barello S., Bonanomi A., Riva G. (2017). Factors affecting patients’ online health information-seeking behaviours: The role of the Patient Health Engagement (PHE) Model. Patient Educ. Couns..

[8] Coulter A. (2012). Leadership for Patient Engagement.

[9] Hochhalter A.K., Song J., Rush J., Sklar L., Stevens A. (2010). Making the Most of Your Healthcare intervention for older adults with multiple chronic illnesses. Patient Educ. Couns..

[10] Greene J., Hibbard J.H., Sacks R., Overton V., Parrotta C.D. (2015). When patient activation levels change, health outcomes and costs change, too. Health Aff..

[11] Wagner P.J., Dias J., Howard S., Kintziger K.W., Hudson M.F., Seol Y.H., Sodomka P. (2012). Personal health records and hypertension control: A randomized trial. J. Am. Med. Inform. Assoc..

[12] Ocloo J., Matthews R. (2016). From tokenism to empowerment: Progressing patient and public involvement in healthcare improvement. BMJ Qual. Saf..

[13] Graffigna G. (2016). Promoting Patient Engagement and Participation for Effective Healthcare Reform.

[14] Castro E.M., Van Regenmortel T., Vanhaecht K., Sermeus W., Van Hecke A. (2016). Patient empowerment, patient participation and patient-centeredness in hospital care: A concept analysis based on a literature review. Patient Educ. Couns..

[15] Elwyn G., Crowe S., Fenton M., Firkins L., Versnel J., Walker S., Cook I., Holgate S., Higgins B., Gelder C. (2010). Identifying and prioritizing uncertainties: Patient and clinician engagement in the identification of research questions. J. Eval. Clin. Pract..

[16] Barello S., Graffigna G., Vegni E., Savarese M., Lombardi F., Bosio A.C. (2015). “Engage me in taking care of my heart”: A grounded theory study on patient-cardiologist relationship in the hospital management of heart failure. BMJ Open.

[17] Greenhalgh T. (2009). Patient and public involvement in chronic illness: Beyond the expert patient. BMJ.

[18] Fox N.J., Ward K.J., O’Rourke A.J. (2005). The “expert patient”: Empowerment or medical dominance? The case of weight loss, pharmaceutical drugs and the Internet. Soc. Sci. Med..

[19] Shaw J., Baker M. (2004). “Expert patient”—Dream or nightmare?. BMJ.

[20] Parsons S., Winterbottom A., Cross P., Redding D. (2010). The Quality of Patient Engagement and Involvement in Primary Care.

[21] Hardyman W., Daunt K.L., Kitchener M. (2015). Value Co-Creation through Patient Engagement in Health Care: A micro-level approach and research agenda. Public Manag. Rev..

[22] Weil A.R. (2016). The Patient Engagement Imperative. Health Aff..

[23] Carr A.J., Gibson B., Robinson P.G. (2001). Is quality of life determined by expectations or experience?. BMJ.

[24] Gibson A., Britten N., Lynch J. (2012). Theoretical directions for an emancipatory concept of patient and public involvement. Health.

[25] Shoemaker S.J., de Ramalho Oliveira D., Alves M., Ekstrand M. (2011). The medication experience: Preliminary evidence of its value for patient education and counseling on chronic medications. Patient Educ. Couns..

[26] Fontaine P., Whitebird R., Solberg L., Tillema J., Smithson A., Crabtree B.F. (2014). Minnesota’s Early Experience with Medical Home Implementation: Viewpoints from the Front Lines. J. Gen. Intern. Med..

[27] Rathert C., Brandt J., Williams E.S. (2012). Putting the “patient” in patient safety: A qualitative study of consumer experiences. Health Expect..

[28] Hibbard J.H., Greene J. (2013). What the evidence shows about patient activation: Better health outcomes and care experiences; fewer data on costs. Health Aff..

[29] Manary M.P., Boulding W., Staelin R., Glickman S.W. (2013). The Patient Experience and Health Outcomes. N. Engl. J. Med..

[30] Barello S., Graffigna G. (2016). Engagement-sensitive decision making: Training doctors to sustain patient engagement in medical consultations. Patient Engagement: A Consumer-Centered Model to Innovate Healthcare.

[31] Williams N., Fleming C. (2011). Consumer and Provider Perspectives on Shared Decision Making: A Systematic Review of the Peer-Reviewed Literature.

[32] Légaré F. (2013). Shared decision making: Moving from theorization to applied research and hopefully to clinical practice. Patient Educ. Couns..

[33] Makoul G., Clayman M.L. (2006). An integrative model of shared decision making in medical encounters. Patient Educ. Couns..

[34] Elwyn G., Lloyd A., May C., van der Weijden T., Stiggelbout A., Edwards A., Frosch D.L., Rapley T., Barr P., Walsh T. (2014). Collaborative deliberation: A model for patient care. Patient Educ. Couns..

[35] Grande S.W., Faber M.J., Durand M.A., Thompson R., Elwyn G. (2014). A classification model of patient engagement methods and assessment of their feasibility in real-world settings. Patient Educ. Couns..

[36] Elwyn G., Frosch M.L., Thomson R., Joseph-Williams N., Lloyd A., Kinnersley P., Cording E., Tomson D., Dodd C., Rollnick S. (2012). Shared decision making: A model for clinical practice. J. Gen. Intern. Med..

[37] Haase J.E., Britt T., Coward D.D., Leidy N.K., Penn P.E. (1992). Simultaneous Concept Analysis of Spiritual Perspective, Hope, Acceptance and Self-transcendence. Image J. Nurs. Sch..

[38] Walker L.O., Avant K.C. (2011). Strategies for Theory Construction in Nursing.

[39] Graffigna G., Barello S., Riva G., Castelnuovo G., Corbo M., Coppola L., Daverio G., Fauci A., Iannone P., Ricciardi W. (2017). Recommandation for patient engagement promotion in care and cure for chronic conditions. Recenti Prog. Med..

[40] Menichettij C., Bussolind G., Barello S., Graffigna G., Corbo M., Coppola L., Daverio G., Fauci A., Iannone P., Ricciardi W. (2016). Engaging older people in healthy and active lifestyles: A systematic review. Ageing Soc..

[41] Menichetti J., Graffigna G. (2016). How older citizens engage in their health promotion: A qualitative research-driven taxonomy of experiences and meanings. BMJ Open.

[42] Menichetti J., Graffigna G., Steinsbekk A. (2018). What are the contents of patient engagement interventions for older adults? A systematic review of randomized controlled trials. Patient Educ. Couns..

[43] Hibbard J.H., Stockard J., Mahoney E.R., Tusler M. (2004). Development of the Patient Activation Measure (PAM): Conceptualizing and Measuring Activation in Patients and Consumers. Health Serv. Res.

[44] Carman K.L., Dardess P., Maurer M., Sofaer S., Adams K., Bechtel C., Sweeney J. (2013). Patient and family engagement: A framework for understanding the elements and developing interventions and policies. Health Aff..

[45] Forbat L., Cayless S., Knighting K., Cornwell J., Kearney N. (2009). Engaging patients in health care: An empirical study of the role of engagement on attitudes and action. Patient Educ. Couns..

[46] Graffigna G., Barello S., Bonanomi A. (2017). The role of Patient Health Engagement model (PHE-model) in affecting patient activation and medication adherence: A structural equation model. PLoS ONE.

[47] Dunston R., Lee A., Boud D., Brodie P., Chiarella M. (2009). Co-Production and Health System Reform—From Re-Imagining to Re-Making. Aust. J. Public Adm..

[48] McBride T., Korczak V. (2007). Community consultation and engagement in health care reform. Aust. Health Rev..

[49] Duke C.C., Lynch W.D., Smith B., Winstanley J. (2015). Validity of a New Patient Engagement Measure: The Altarum Consumer Engagement (ACE) Measure^TM^. Patient.

[50] Graffigna G., Barello S., Bonanomi A., Lozza E. (2015). Measuring patient engagement: Development and psychometric properties of the patient health engagement (PHE) scale. Front. Psychol..

[51] Battersby M.W., Ask A., Reece M.M., Markwick M.J., Collins J.P. (2003). The Partners in Health scale: The development and psychometric properties of a generic assessment scale for chronic condition self-management. Aust. J. Prim. Health.

[52] Howie J.G.R., Heaney D.J., Maxwell M., Walker J.J. (1998). A comparison of a Patient Enablement Instrument (PEI) against two established satisfaction scales as an outcome measure of primary care consultations. Fam. Pract..

[53] Cramm J.M., Strating M.M.H., de Vreede P.L., Steverink N., Nieboer A.P. (2012). Validation of the self-management ability scale (SMAS) and development and validation of a shorter scale (SMAS-S) among older patients shortly after hospitalisation. Health Qual. Life Outcomes.

[54] Lorig K., Chastain R.L., Ung E., Shoor S., Holman H.R. (1989). Development and evaluation of a scale to measure perceived self-efficacy in people with arthritis. Arthritis Rheum..

[55] Kortte K.B., Falk L.D., Castillo R.C., Johnson-Greene D., Wegener S.T. (2007). The Hopkins Rehabilitation Engagement Rating Scale: Development and Psychometric Properties. Arch. Phys. Med. Rehabil..

[56] Zwicker D. (2010). Preparedness for caregiving scale. Mov. Disord..

[57] DeCamp L.R., Leifheit K., Shah H., Valenzuela-Araujo D., Sloand E., Polk S., Cheng T.L. (2016). Cross-cultural validation of the parent-patient activation measure in low income Spanish- and English-speaking parents. Patient Educ. Couns..

[58] Hibbard J.H., Collins P.A., Mahoney E., Baker L.H. (2010). The development and testing of a measure assessing clinician beliefs about patient self-management. Health Expect..

[59] Greene J., Sacks R.M., Hibbard J.H., Overton V. (2016). How much do clinicians support patient self-management? The development of a measure to assess clinician self-management support. Healthcare.

[60] Linden A., Butterworth S.W., Prochaska J.O. (2010). Motivational interviewing-based health coaching as a chronic care intervention. J. Eval. Clin. Pract..

[61] Leach E., Cornwell P., Fleming J., Haines T. (2010). Patient centered goal-setting in a subacute rehabilitation setting. Disabil. Rehabil..

[62] Bigi S. (2016). Communication skills for patient engagement: Argumentation competencies as means to prevent or limit reactance arousal, with an example from the Italian healthcare system. Front. Psychol..

[63] Stein T., Frankel R.M., Krupat E. (2005). Enhancing clinician communication skills in a large healthcare organization: A longitudinal case study. Patient Educ. Couns..

[64] Deen D., Lu W.H., Rothstein D., Santana L., Gold M.R. (2011). Asking questions: The effect of a brief intervention in community health centers on patient activation. Patient Educ. Couns..

[65] Knops A.M., Legemate D.A., Goossens A., Bossuyt P.M.M., Ubbink D.T. (2013). Decision aids for patients facing a surgical treatment decision: A systematic review and meta-analysis. Ann. Surg..

[66] Fromer L. (2011). Implementing chronic care for COPD: Planned visits, care coordination, and patient empowerment for improved outcomes. Int. J. COPD.

