# Supplementary material for: Italian Consensus Statement on Patient Engagement in Chronic Care: Process and Outcomes
Source: Int J Environ Res Public Health. 2020 Jun 11;17(11):4167. doi: 10.3390/ijerph17114167 (PMC7312656; doi:10.3390/ijerph17114167)
Supplement: Supplementary file 1 [file ijerph-17-04167-s001.pdf]

Table S1. Glossary of the key concepts of participatory medicine and their conceptual relationship with engagement.

Concept	Definition	Differences/ Connections with Engagement
**Activation (i.e., the Person's Behavioral Activation)**	Level of knowledge, skill and faith of the individual regarding his ability to manage his treatment and interacting with the welfare system.	It mainly focuses on the behavioural dimension of the individual's experience in his exchange with the healthcare professionals and the health and social services and in self-management.
**Empowerment (i.e., the Feeling of Power and Control over One's Own Health Management)**	It concerns a mental state that the individual experiences at the moment when he perceives himself as having control and power on the current state of his health and treatments.	If empowerment focuses on the individual within a relationship, Engagement, suggesting a systematic view of the individual in his healthcare process in which he is the main protagonist, focuses on the relationship itself.
**Compliance (i.e., the Person's Ability to Follow Clinical Prescriptions)**	Degree to which a person's behaviour (assumption of pharmaceuticals, adherence to diet plan, changes in lifestyle) coincides with the clinical recommendations given (Consensus Conference on Narrative Medicine, 2015 p. 23). Successively, the term 'adherence' included in his own definition, a conceptual change by talking about the person's participation in the planning and treatment actualization. However, talking about compliance or adherence implies an evaluating position of the healthcare professional towards a person, which is then defined as "more or less good", "more or less capable" of responding/adhering in an adequate way to the clinical and care requests.	The concept of Engagement itself goes far beyond the specific evaluation of the individual's behaviour and attitude towards the clinician's prescriptions. To talk about Engagement means to adopt a new strongly democratic vision of the exchange process between the healthcare professional and the individual and between the demand and supply of social and healthcare practises, in which the various actors, their subjectivity and their different skills are all part of a synergic system so as to promote positive dialogue and use of the healthcare service in its complexity.
**Therapeutic Education**	Therapeutic education should allow the person to acquire and maintain the skills and competences that help him live in an optimal way with his illness. Education implies an organized activity of sensitization, information, learning of self-management concerning the illness, the prescribed treatment, the therapies, the cure and hospital context, the information regarding the organization and the health and illness behaviours. The goal of this education is to help people and their families to understand the illness and the treatment, cooperate with healthcare professionals, live in a healthier way and maintain or improve their quality of life. Therapeutic education trains the ill patient to acquire an adequate knowledge, a know-how and know-how-to-be in order to reach a balance between his life and the optimal control of his illness […] it's a continuous process that is an integrating part of healthcare and assistance	The concept of Engagement includes the one of therapeutic education by considering it as one of the fundamental instruments to allow the person and his informal caregivers to acquire the knowledges and competences needed to have an active role in the clinical care journey.
**Health Literacy**	Set of personal cognitive and social skills that determine the individual's ability to manage the illness, that make him capable of accessing, understanding and using	The concept of health literacy refers to the person's knowledges and his abilities to use information. For this reason it represents

	the information so as to promote and preserve his health (Nutbeam, 2000)	one of the levers that contribute in promoting Engagement but not in an exhaustive way.
**Narrative Medicine**	Narrative medicine is based on the assumption that narration is the "key element to acquire, understand and integrate the different points of view of those who intervene in the illness and the care process" (Consensus Conference on Narrative Medicine, 2015, p.13). In conformity with these epistemological premises, Narrative Medicine can be considered a clinical care intervention methodology that focuses on a specific communicative and relational strategy between individuals and the healthcare team.	The relationship between Narrative Medicine and Engagement is evident, as Narrative Medicine can be considered as one of the strategies used to increase subjective participation perception and the individual's sense of protagonism in the care process. Narrative Medicine is to be considered as a possible strategy or instrument of intervention for the humanization of the health and social service system, of which Engagement can be one of the goals to strive for.
**Patient-Centered Medicine**	It's a method of clinical intervention based on the bio-psycho-social paradigm. It's a new model for clinical practice orientation that doesn't want to be an alternative to the traditional model but that expands its boundaries whilst keeping its scientific value (Moja & Vegni, 2000).	Patient-centered medicine is a model that directs the clinician's work in the light of a better understanding and taking care of the person's needs and illness experiences. It's the fundamental orientation needed to advance the possibility of Engagement in the individual and to direct sensitivity and opening of the professional to the proactive role of the person.
**Personalized Medicine**	Personalized Medicine is a form of clinical intervention that uses generic type of informations, linked to the environmental influence and the life style of the individual to suggest prevention and treatment interventions that are highly personalized (Swan M., 2012)	The concept of personalized medicine doesn't necessarily imply patient Engagement in the care process as it more specifically refers to the definition of a therapeutic plan that "fits" on the person's clinical needs. The measurements of Engagement level, however, can be an ulterior diagnostic piece for a truly personalized medicine.
**Participatory Medicine**	A cooperative assistance model that promotes the active involvement of the people, the professionals, caregiver and other actors through the continuative process of undertaking of all the aspects that deal with the individual's health. A movement through which people are pushed to change from being a passenger to being "co-pilots" of the choices that concern their health, while the professionals are encouraged and valued in being their partner (*Society for Participatory Medicine*, http://participatorymedicine.org/).	Participatory Medicine offers a welfare model that focuses on the active role of the individual in his care process. Therefore, in this respect participatory medicine represents the proposal of revision of the care models that better suit with the concept of Engagement.
**Expert Patient**	The term "expert patient" was created in 1985 by prof. David Tucket of Cambridge University. An Expert patient is one with experience of his illness and the disease that he is affected with	The definition of "expert patient" denotes the skills and knowledges of the patient on his illness and his psychological experience of it. The expert patient is the prerequisite and not the result of an Engagement which is efficient and functional to the development of a different way of curing oneself/curing, by governing the care process itself.
**Shared Decision Making**	Through shared decision making, doctor and patient cooperate to reach a shared	Shared decision making process is one of the prototypical clinical

	decision regarding the treatment approach after having assessed together the risks and benefits of different diagnostic-therapeutic options, taking into account values, preferences and specific patient's conditions.	setting in which the concept of patient Engagement can be applied. The level of patient Engagement can be considered as a precursor or a *conditio sine qua non* to make a shared decisional process possible.
**Engagement in Research**	The *Patient Centered Research Outcome Institute* (*PCORI*) defines the Engagement of people, their families, healthcare professionals and other significant stakeholders in the research process related to pharmacological innovation or in the design and realization phases of the different services.	Research is one of the settings in which the concept of Engagement can be applied.

Table S2. Glossary of the main approaches and methodologies for promoting engagement.

Level of Intervention	Approach/ Methodology	Definition
Patient/Caregiver	**Motivational Interviewing**	Motivational interviewing is a mean to help the person change his problematic behaviors (for example, substance abuse), by becoming more aware and act on the motivations underlying them.
	**Therapeutic Education**	Therapeutic Education allows the person to acquire and maintain the skills and competences that help him live with his illness, in the most effective way. Education includes organized activities of sensitization, information, learning of self-management regarding the illness, prescribed treatment, therapies, hospital and cure context and the information regarding the organization and the health and illness behaviors. It's designed to help people and their family understand the illness and the treatment, cooperate with the healthcare professionals, live in a healthier way and maintain or improve their quality of life. Therapeutic education trains the individual with an illness so that he can acquire an adequate "knowledge, know-how, and know-how-to-be" to reach a balance between his life and the optimal control of the health condition. This is a continuous process that is an integrating part of the curing process.
	**Peer Education**	In peer education, ad adequately trained individual (peer trainer) launches training activities with other peers, which are people with roughly the same age, working condition, gender, status, culture or life experiences. Peer education is part of the "social mediation" learning methods along with cooperative learning and peer tutoring. As a method of intervention in the field of health promotion and more in general in prevention of at-risk behaviors, it is –according to some authors- more appropriately translated to "peer-prevention". These educational activities aim to strengthen, amongst peers, knowledge, attitudes, and skills that allow the making of responsible choices that are aware of one's own health. Peer education aims to broaden the window of actions that a person has, and to help the person develop critical thinking on the behaviors that could impede his physical, psychological and social wellbeing and a good quality of life.
**Patient/Caregiver**	**Mindfulness**	The theory of mindfulness starts from the rediscovery of methods of psychological changes shaped around intuitive means of self-knowledge, in addition to discursive and verbal methods of problem-solving. Mindfulness promotes experiences that welcome the present, that allow for a broader and finer understanding of difficulties and tolerance of negative emotions and perceptions as experiences to include and overcome with equanimity in one's own existential journey.
**Healthcare Professionals**	**Inter Professional Education (IPE)**	Educational and training approach dedicated to both university students and to professional's lifelong training that values an inter-professional exchange, active learning that is situated beyond multi-professional and multi-disciplinary collaboration.
	**Problem Based Learning (PBL)**	Problem-based learning is a teaching method in which a problem is the starting point of the learning process. PBL belongs to the category of teaching methods that are learner-centered.
	**Expert Patients**	The term "expert patient" was created in 1985 by prof. David Tucket of Cambridge University. An Expert

		patient is one with experience of his illness and the disease that he is affected with.
	**Expansive Learning**	Expansive learning is defined by Engeström as follows: 'Expansive learning is initiated when some individuals involved in a collective activity take the action of transforming an activity system through reconceptualization of the object and the motive of activity embracing a radically wider horizon of possibilities than in the previous mode of activity
**Healthcare Organizations**	**Personalized Treatment Plans**	Set of prescriptions, requirements and clinical care actions that are necessary for a better taken care of of the person, personalized based on his clinical, assistive and social needs.
	**Service Co-Design/Co-Production of Services**	Prospective that focuses on the involvement of all the significant stakeholders in the definition, design, implementation and assessment of services. Namely, a process of co-production through the participation of the citizens in the realization of the provision of services of public utility, in order to guarantee the construction of a welfare characterized by high levels of quality, which is also democratic and qualifying.
**Society**	**Social Marketing Campaigns**	Implementation of concepts and technologies of marketing and other disciplines to reach behavioral goals aimed at improving individual/collective well-being and to contribute in decreasing social inequalities, in the context of the policies of the community in question.
